# Water Adsorption in Metal–Organic Frameworks: Characteristics, Mechanisms, and Structure–Property Relationships

**DOI:** 10.1021/jacs.5c10686

**Published:** 2025-09-10

**Authors:** Shiue-Min Shih, Li-Chiang Lin

**Affiliations:** a Department of Chemical Engineering, 33561 National Taiwan University, Taipei 106319, Taiwan; b William G. Lowrie Department of Chemical and Biomolecular Engineering, The Ohio State University, Columbus, Ohio 43210, United States

## Abstract

To address the increasingly limited water availability, using metal–organic frameworks (MOFs) to capture atmospheric water vapor as usable resources has emerged as a promising strategy. The adsorption characteristics of MOFs as well as their step pressure (i.e., the pressure when the steep rise in water uptake, if any, occurs) directly influence the amount of collectable water and thus their harvesting effectiveness. To date, a comprehensive understanding of water adsorption behaviors and how they are influenced by MOF properties remains lacking. This study addresses this gap by systematically investigating water adsorption in >200 strategically selected MOFs with diverse features. The results demonstrate that water adsorption in MOFs is highly complex, revealing various subtypes of both non-S-shaped and S-shaped isotherms. Moreover, among S-shaped isotherms, distinct phase behaviors that, in turn, govern different adsorption mechanisms and hydrogen-bonding environments are identified, as evidenced by analyses of their macrostate probability distributions (MPDs), thermodynamic stability limits, and grand potential free energy landscapes. Further structure–property analyses also uncover key design principles: A moderate heat of adsorption is critical for enabling S-shaped isotherms, while the size of pores critically modulates the steepness of the adsorption step by controlling the phase transition nature of confined water. The density and, more importantly, the uniformity of adsorption sites are found as well to dictate the step pressure. Overall, this study provides a comprehensive overview of diverse adsorption behaviors in MOFs as well as informs general design principles for the development of advanced adsorbents for atmospheric water harvesting.

## Graphical abstract

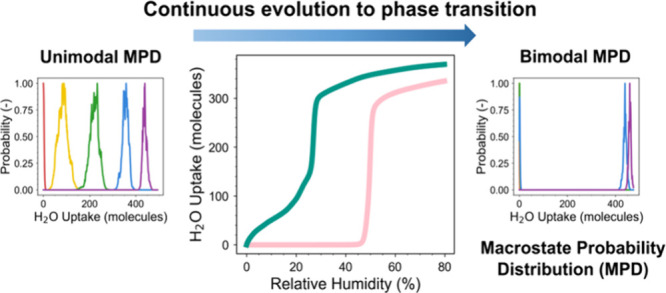

## 1 Introduction

Water scarcity has become an increasingly pressing challenge of our time. Approximately 4 billion people are currently experiencing water shortages,^1^ and this number is projected to be 4.8 to 5.7 billion people by 2050,^2^ with most of them living in inland regions with arid climates.^3^ This growing crisis underscores the urgent need to seek unconventional alternatives to produce freshwater. Among emerging solutions, adsorbent-assisted atmospheric water harvesting (AWH) has drawn considerable attention. This process captures atmospheric water vapor, a source that is not limited by geographic or climatic constraints,^4^ and converts it into liquid water via a temperature or pressure swing adsorption (TSA/PSA) cycle.^5,6^ In a typical AWH cycle, water vapor is adsorbed in the adsorbent during the night with lower temperature and higher relative humidity (RH). When the temperature rises or ambient RH drops during the daytime, the adsorbed water is desorbed and subsequently condensed into liquid form (Figure 1a). This inherent alignment with daily temperature and RH shifts significantly reduces the energy required for operation, making this process highly energy-efficient.^7–9^ Alternatively, thermoelectric coolers can also be employed to drive the process.^10^

**Figure 1:**
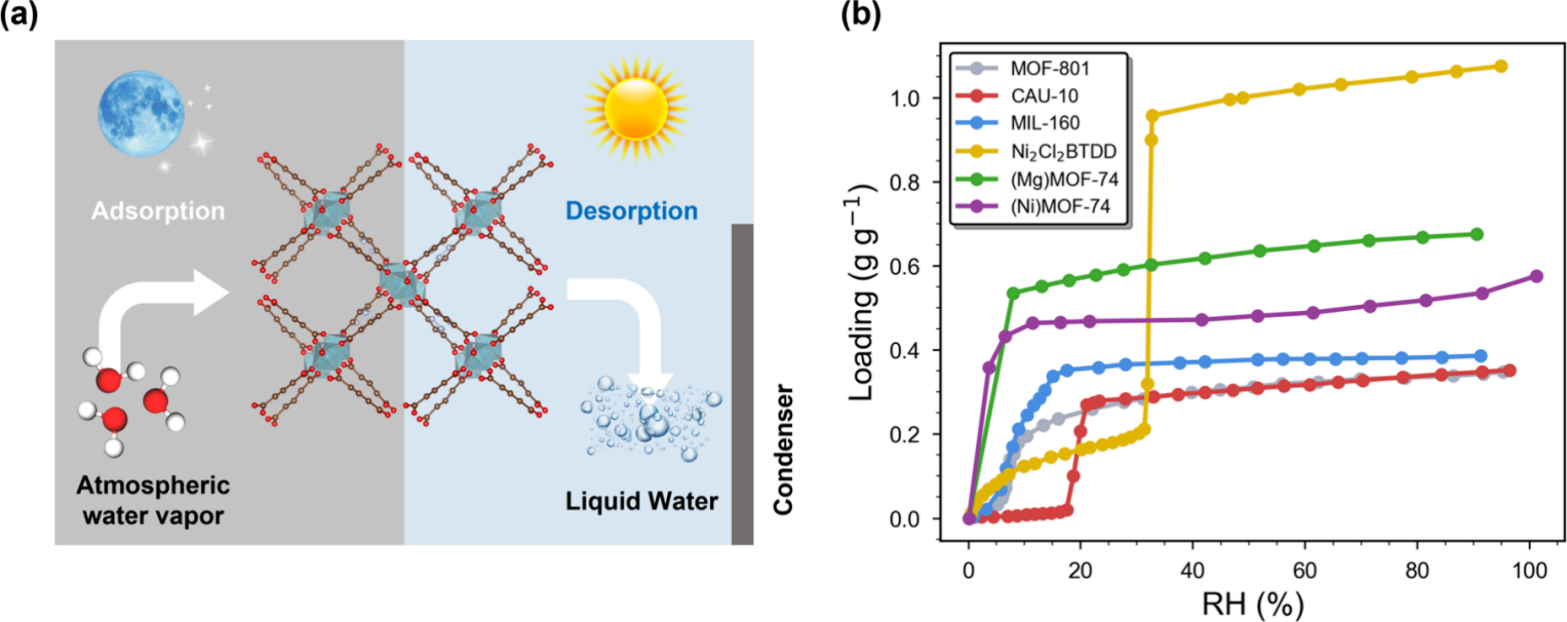
(a) Schematic illustration of the atmospheric water harvesting (AWH) process using metal–organic frameworks (MOFs) as the adsorbent. During the night, the MOF captures water vapor from the atmosphere. As the temperature rises with decreased humidity during the day, the adsorbed water is released and subsequently condensed into liquid. (b) Diverse water adsorption isotherms reported in the literature for MOF-801, MIL-160, CAU-10, Ni_2_Cl_2_BTDD, (Mg)­MOF-74, and (Ni)­MOF-74. Data for MOF-801 are adapted from ref 9; MIL-160 and CAU-10 from ref 22; Ni_2_Cl_2_BTDD from ref 21; and (Mg)­MOF-74 and (Ni)­MOF-74 from ref 20. RH refers to relative humidity.

The effectiveness of AWH hinges on the adsorption properties of the employed adsorbent. A key performance indicator is the deliverable capacity, i.e., the amount of water that can be reversibly stored and released per each adsorption/desorption cycle. As would be intuitively anticipated, adsorbents exhibiting S-shaped adsorption isotherms, characterized by a steep rise in water uptake within a narrow pressure window, are desired as such materials may be more likely to offer a higher deliverable capacity.^8^ In addition, water condensation must occur within the adsorption–desorption pressure range, particularly within the 10–30% RH window for arid regions.^11^ This pressure is typically referred to as the “step pressure”. To date, numerous materials have been evaluated for their potential in AWH, including silica gels,^12^ zeolites,^13^ and metal–organic frameworks (MOFs).^14,15^ Silica gels are commercially available, are nontoxic, and exhibit fast adsorption kinetics with low regeneration temperatures.^16^ However, their rather limited water uptake at a low RH reduces their effectiveness in AWH applications.^17^ Zeolites, on the other hand, are known for their high water affinity,^18^ but they generally suffer from high regeneration temperature^17^ as well as slow kinetics.^19^ Compared to the above sorbents, MOFs exhibit higher water capacity and lower desorption energy.^20^ Their high porosity, tunable hydrophilicity, and diverse structures endow them with great potential for efficient AWH.^17^

Recent research focus has increasingly turned toward exploring and designing MOFs as AWH adsorbents. Prior studies have demonstrated the diverse water adsorption characteristics in various well-known MOFs, as illustrated in Figure 1b. For example, MOF-801 exhibits a classic S-shaped isotherm initiated by water adsorption at hydrophilic −OH groups.^9^ Ni_2_X_2_BTDD also displays an S-shaped isotherm, where its step pressure can be tuned through anion exchange.^21^ MOF-74 series features Langmuir-like isotherms attributed to the strong water adsorption onto open metal sites.^20^ Between MIL-160 and CAU-10, the former, though isostructural, exhibits a lower step pressure and a more gradual adsorption profile.^22^ While these studies have advanced our understanding of water adsorption in MOFs, their focus on a small set of materials offers rather limited insights, both macroscopically and microscopically, into the overall adsorption picture. In addition, while a recent study by Oppenheim and Dincǎ successfully established a quantitative relationship among water sorbent capacity, critical RH for capillary condensation, and pore composition and topology,^23^ atomistic-level understandings on the formation of S-shaped isotherms, the value of the critical RH, and how they are influenced by the structural features are still missing. Overall, a comparative investigation across a diverse set of MOFs for a systematic and comprehensive understanding remains critically needed for their future rational selection and optimization.

To address this knowledge gap, by employing state-of-the-art Monte Carlo simulations and thermodynamic analyses, this study systematically explores water adsorption characteristics in diverse MOFs. Specifically, the adsorption of water in >200 diverse MOFs selected from the 2019 computational-ready experimental (CoRE) MOF database^24^ is investigated. Our results, for the first time, reveal seven distinct types of isotherm characteristics. Moreover, through analyses based on their macrostate probability distribution (MPD), the occurrence of phase separation is found to play a key role, leading to distinct water adsorption mechanisms and hydrogen-bonding environments. Subsequently, with the large data set at our disposal, we examine the structure–property relationship, including the emergence of S-shaped characteristics and step pressure positions. S-shaped isotherms with a sharp uptake increase are more commonly observed in MOFs with larger pore sizes, higher channel dimensionality, and lower hydrophilicity, while isotherms with lower step pressures tend to occur in structures with more homogeneous adsorption sites.

## 2 Computational Details

In this study, MOFs are strategically selected from the 2019 computational-ready experimental (CoRE) MOF database.^24^ The database employed herein is further curated as reported in Wang et al.^25^ to remove potentially problematic structures. Specifically, this study focuses on MOFs of relatively moderate heat of adsorption (HoA) determined under the infinite dilution condition with a value ranging from ∼40 to ∼60 kJ/mol. Moreover, for a more focused comparison, three subgroups of MOFs are studied: 75 MOFs with HoA to be ∼40 kJ/mol (i.e., 38–41 kJ/mol), ∼50 kJ/mol (i.e., 48–51 kJ/mol), and ∼60 kJ/mol (i.e., 58–61 kJ/mol), leading to a total of 225 MOFs. This approach enables us to compare not only structures with distinct HoA values but also those with nearly identical HoA but distinct geometric and chemical features. To enable such a selection, the HoA of water under infinite dilution for MOFs studied in this work is first calculated using the well-established Widom particle insertion approach.^26^ We note that the focus on this selected range of HoA may be considered somewhat arbitrary but should in fact be deemed reasonable, provided that those with a too small or too large HoA will result in a too low uptake or make the regeneration of adsorbents challenging.^25^ A list of all studied 225 MOFs is summarized and provided as a part of the Supporting Information (SI), along with a variety of their properties obtained in this work, as will be shown in later sections. Their MOF refcode can be referenced through both the CCDC Web site^27^ and the MOFXDB database^28^ to source back to their original paper as well as their common name, if any.

Subsequently, to compute their water adsorption isotherms, while grand canonical Monte Carlo (GCMC) simulations have been nearly exclusively employed for this purpose, they have been demonstrated to converge very slowly and may be problematic.^29^ To this end, the NVT+W simulations^30,31^ first reported by Smit and co-workers, a variant of the flat histogram Monte Carlo approaches, are employed herein. The NVT+W method equally samples each accessible loading macrostate (i.e., number of molecules (*N*) in a simulation supercell), ranging from *N* = 0 to *N*_max_ where *N*_max_ is the saturated loading. Specifically, Widom insertion and deletion trials are performed on the fly under a given chemical potential μ during the canonical simulations for each *N* value at fixed volume *V* and temperature *T*. The statistics of these trial moves are accumulated in a collection matrix (C-matrix) to obtain the MPD, Π­(*N*; μ, *V*, *T*). The resulting MPD can be reweighted to any other chemical potentials μ′, resulting in a complete adsorption isotherm at *T*. A schematic workflow of the NVT+W method is illustrated in Figure 2. The open-source RASPA package^32^ with in-house modifications is used in this study. More details can be found in SI Sections 1.1 and 1.2, and the readers are also referred to several seminal studies reported in the literature.^29–31^

**Figure 2:**
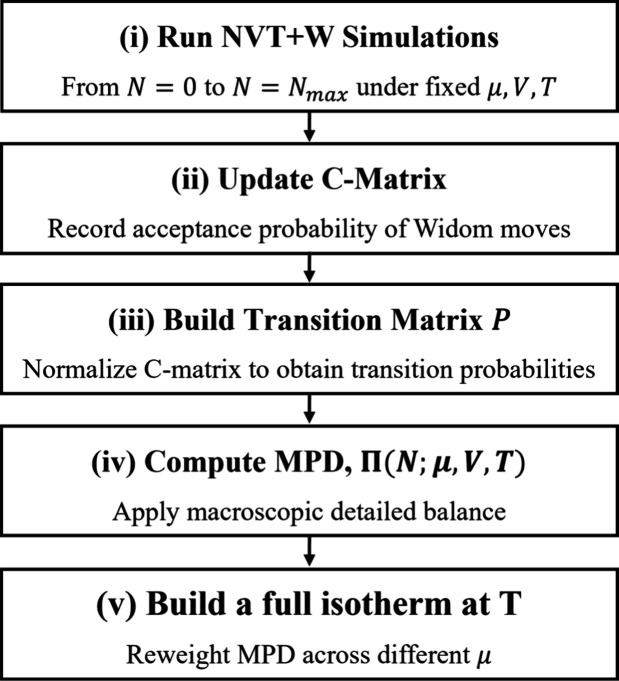
Schematic flowchart of the employment of NVT+W simulations to determine adsorption isotherms in MOFs. The procedure consists of the following steps: (i) Conduct NVT+W simulations for each possible macrostate *N*, ranging from *N* = 0 to *N* = *N*_max_, under a fixed μ, *V*, and *T*. Herein, μ corresponds to 20% RH and *T* = 298 K. (ii) Update C-matrix by recording the attempted Widom insertion/deletion moves for their acceptance probability per grand canonical ensemble. (iii) Construct the macrostate transition probability matrix *P* by normalizing the C-matrix. (iv) Determine macrostate probability distribution (MPD), Π­(*N*; μ, *V*, *T*), via detailed balance between macrostates. (v) Reweight MPD to yield a full isotherm.

All the NVT+W simulations are conducted at 20% RH (i.e., reference μ) and 298 K. *N*_max_ is estimated as 1.1 times the geometric saturation loading, which is obtained by multiplying the void volume of the adsorbent determined by Zeo++ ^33,34^ with the liquid water density. Such chosen *N*_max_ is found to be sufficiently large, ensuring all relevant macrostates have been properly sampled (see SI Section 1.4 for more details). In these calculations, intermolecular interactions are computed using the pairwise sum of 12–6 Lennard-Jones (L-J) potentials and Coulombic interactions. L-J parameters are sourced from DREIDING,^35^ with those that are not available to be instead adopted from UFF.^36^ The partial charges for the framework atoms are assigned by the multilayer connectivity-based atom contribution (m-CBAC) method.^37^ Water molecules are modeled using the TIP4P-EW model.^38^ Details of all adopted parameters can be found in SI Section 1.3. L-J interactions are truncated and shifted at a cutoff radius of 12 Å, while long-range Coulombic interactions are calculated using the Ewald summation method^39^ with a precision of 10^–6^. In addition, MOF frameworks are treated as rigid. We note that the use of generic force fields with rigid framework assumptions has been widely adopted in numerous seminal studies as reported in the literature,^40–43^ particularly for large-scale computational material explorations as conducted herein. While recent advances in machine learning potentials with the consideration of the framework flexibility, such as the study of Goeminne and Van Speybroeck,^44^ provide tremendous opportunities for more accurate simulation predictions, their associated computational costs, in terms of both model training and their applications in Monte Carlo calculations, to investigate a large number of structures are simply prohibitive. Therefore, although the adopted, rather traditional, approach may have certain limitations, it represents the most viable one to be employed and the resulting outcomes should still be deemed physically sound. It is important to also note that the saturation pressure (*P*_0_) of water should correspond to the employed model (i.e., TIP4P-EW) rather than experimental data, according to the suggestion by Datar et al.^45^ As the TIP4P-EW model has a saturation pressure of 986 Pa,^45,46^ 20% RH corresponds to 197 Pa. All simulations are conducted until convergence as detailed in SI Section 1.4.

## 3 Results and Discussion

This section first presents the complex water adsorption isotherms observed from 225 MOFs studied herein. The underlying mechanisms distinguishing these isotherms are then analyzed through the MPD, thermodynamic stability limits, grand potential free energy profiles, and visualizations on the spatial distribution of adsorption sites. Subsequently, key structural–property relationships are examined to elucidate how the intrinsic structural and chemical characteristics of MOFs influence their water adsorption. Finally, factors governing the step pressure are investigated.

### 3.1 Diverse Adsorption Characteristics

A striking diversity of water adsorption isotherms is observed across the studied MOFs, revealing seven distinct types (Figure 3). The seven distinct isotherms can be primarily classified into non-S-shaped (i.e., denoted as “N–S”) and S-shaped, where each contains multiple subtypes. We note that the isotherm classification presented herein is through visual inspections coupled with MPD analyses. For the latter, as will be discussed later, clear distinctions in MPDs exist between different subtypes of S-shaped isotherms. Figure 3a illustrates three representative subtypes of the N–S isotherms. N–S­(I) shows a steep uptake at very low RH and demonstrates early saturation, where adsorption is dominated by strong MOF–water interactions at primary adsorption sites.^47^ Such adsorption behavior has also been frequently observed experimentally, such as that of the well-known MOF-74 series, as shown in Figure 1b. N–S­(II) features a more gradual and continuous uptake profile over a wide RH range, while N–S­(III) exhibits a slow increase in initial uptake followed by a relatively faster uptake at higher RH. These trends reflect a progressively greater reliance on water–water interactions to drive adsorption. As quantitatively shown in SI Section 2.1, the MOF–water energy contribution decreases most rapidly in N–S­(I) as a function of RH, falling to 60% at just 10% RH. This decay is less pronounced in N–S­(II) and the slowest in N–S­(III); the latter still retains 68% MOF–water contribution even at 100% RH. Collectively, these results suggest that strong hydrophilicity dominates the adsorption in N–S­(I), whereas both N–S­(II) and N–S­(III) increasingly depend on water–water interactions to enhance uptake.

**Figure 3:**
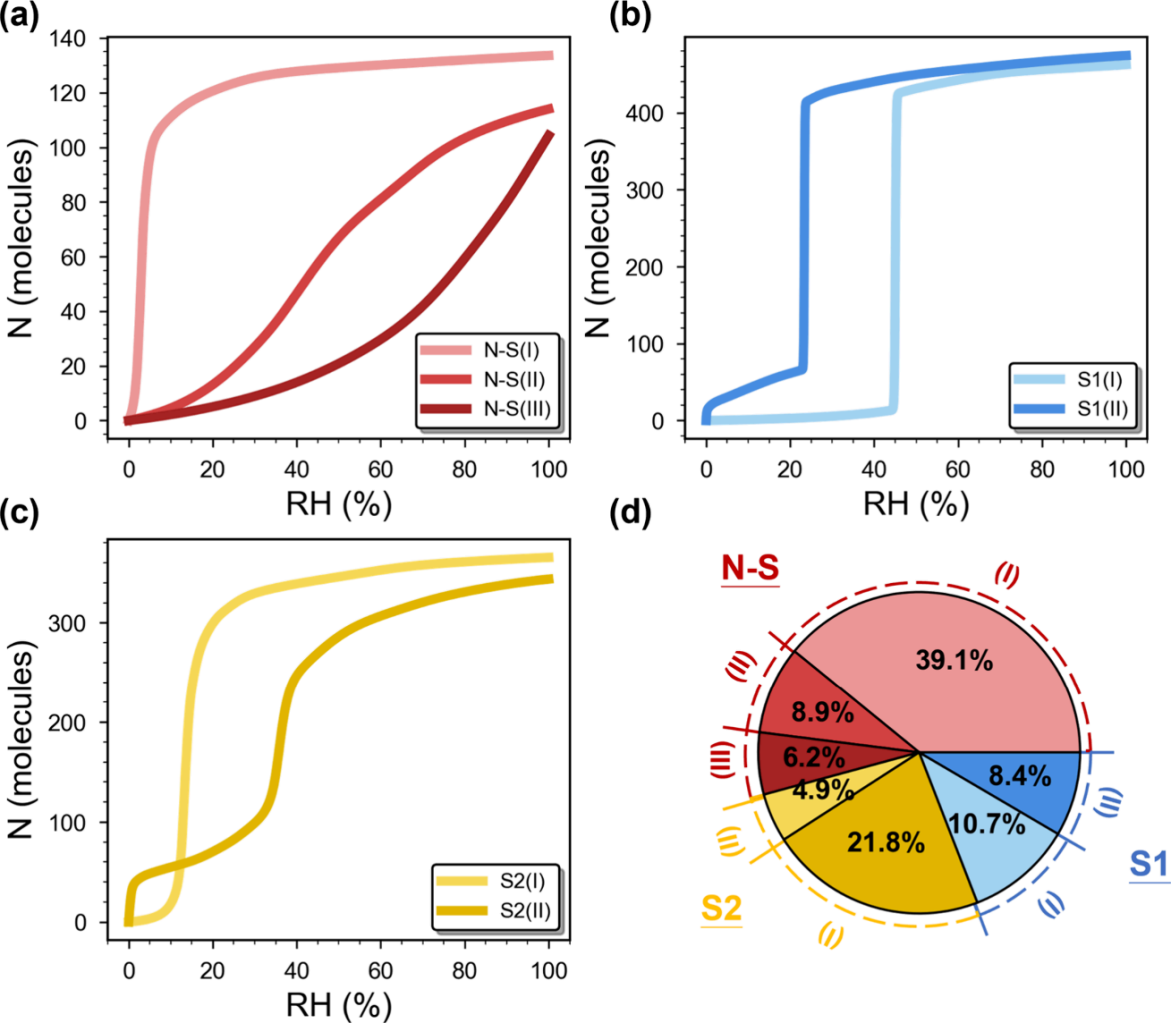
Classification and distribution of N–S and S-shaped isotherms. (a) N–S isotherms and their three subtypes: N–S­(I), N–S­(II), and N–S­(III). (b, c) S-shaped isotherms characterized by a vertical step (i.e., denoted as S1) or a sharp but relatively gradual uptake increase (i.e., denoted as S2). Both S1 and S2 are further categorized into two subtypes (i.e., I and II) per the strength of their initial water adsorption. (d) Distribution of different isotherm types.

Distinct from N–S isotherms, the well-known S-shaped isotherms feature much steeper uptake transitions, indicative of water condensation. Strikingly, two different types as depicted in Figure 3b and Figure 3c (i.e., denoted as S1 and S2, respectively) are observed, which can be visually distinguished by the steepness of their adsorption step. Specifically, the former features a sharp, nearly discontinuous step, whereas the latter exhibits a relatively more gradual water increase. This distinction has in fact been experimentally observed by Hanikel et al.; the authors showed that MOF-303 transitions from a relatively mild to a sharp adsorption step when a hydrophilic functional group is replaced with a more hydrophobic one.^48^ Such a distinction, however, was not discussed. More intriguingly, both types can also show differences in their early-stage adsorption behavior. While some S-shaped isotherms exhibit a very mild initial uptake increase, they may also feature an N–S-like rapid increase in the uptake (i.e., resembling type N–S­(I)). These behaviors are categorized as subtypes (I) and (II), respectively. Although adsorbents of S-shaped characteristics are generally desired in water harvesting as noted above, among these S-shaped isotherms, S1­(I) should be particularly preferred, as such a subtype is more likely to enable a higher deliverable capacity with minimal changes in environmental conditions. Figure 3d shows the distribution of the seven identified isotherm types. The ratio of N–S to S-shaped isotherms approaches 54:46 with N–S­(I) being the most prevalent among N–S subtypes, followed by N–S­(II) and N–S­(III). For S-shaped isotherms, S2­(I) is the most frequently observed subtype, followed by S1­(I), S1­(II), and S2­(II). At this point, it is important to note that for those MOFs being classified to have N–S­(II) or even N–S­(III) isotherms, some of them may possibly show an S-shaped behavior under a *P*/*P*_0_ greater than 1 (i.e., adsorption against liquid water), where *P* represents the pressure. However, provided that the primary focus of this work is for water harvesting, classification is conducted per the isotherm with a *P*/*P*_0_ up to the unity.

The difference between the observed S-shaped isotherms (i.e., S1 vs S2) suggests that their water condensation behavior may be distinct. Interestingly, S1 isotherms are found to exhibit a “bimodal” MPD upon the step pressure, indicating a phase transition between a low-density (vapor-like) and a high-density (liquid-like) state^49^ (Figure 4a). In contrast, S2 isotherms demonstrate a “unimodal” distribution in their corresponding MPD across the entire pressure range (Figure 4d), implying a continuous and thermodynamically stable adsorption process. It should be noted that the abovementioned thermodynamic signature that differentiates S1 and S2 isotherms remains the same regardless of their initial adsorption characteristics (i.e., S1 and S2 isotherms show consistent trends of their MPDs in subtypes (I) and (II). More details can be found in SI Section 2.2). To further decode the molecular-level implications of the observed MPD profiles, their associated thermodynamic stability and grand potential free energy landscapes are examined, both of which can be directly obtained from MPD with details shown in SI Section 2.3. Figure 4b and Figure 4e show their respective profiles of $\frac{\partial^{2}\ln\Pi }{\partial N^{2}}$ at the corresponding step pressure. The second derivative for the S1 isotherm shows a positive value between *N* = 34 and *N* = 363, which evidently defines a thermodynamically unstable window where the system fluctuates between two distinct phases. In contrast, the S2 isotherm exhibits a consistently negative second derivative throughout the entire *N* range, indicating the absence of phase separation (i.e., a single phase). A complementary view is also reflected in the grand potential free energy (*W*) profiles shown in Figure 4c,f. The S1 isotherm displays a characteristic double-well shape at the step pressure with a free energy barrier *W*_b_ ∼100*k*_B_ *T*, which significantly exceeds thermal fluctuations and suppresses spontaneous transitions between the vapor-like and liquid-like states. The S2 isotherm, by contrast, exhibits a single-well free energy profile with only an energy minimum, again indicating that water molecules form a single thermodynamically stable phase within the adsorbent. Overall, S1 isotherms with bimodal MPD undergo a first-order phase transition, while S2 ones with unimodal MPD follow a continuous transformation. To reinforce this connection throughout the discussion and for clarity, we hereafter refer to S1 and S2 isotherms as “bimodal” and “unimodal” isotherms, respectively. At this point, it should be noted that, while isotherms featuring first-order phase transition should be associated with a perfectly vertical step, bimodal isotherms presented herein, such as that in Figure 3b, do not strictly speak features such an infinitely steep rise. This is attributed to the fact that the isotherms presented herein are the so-called “net isotherms”. That is, the bimodal MPD obtained is directly used to compute adsorption uptakes without first decomposing it into the stable and metastable contributions. The readers are also referred to studies of Shen et al.^50^ and Datar et al.^29,51^ for related discussion. Opting for such an approach is primarily for convenience and simplicity considering the large number of structures studied herein. In addition, employing net isotherms also makes the identification of the step pressure, as will be discussed in a later section for bimodal MOFs notably easier.

**Figure 4:**
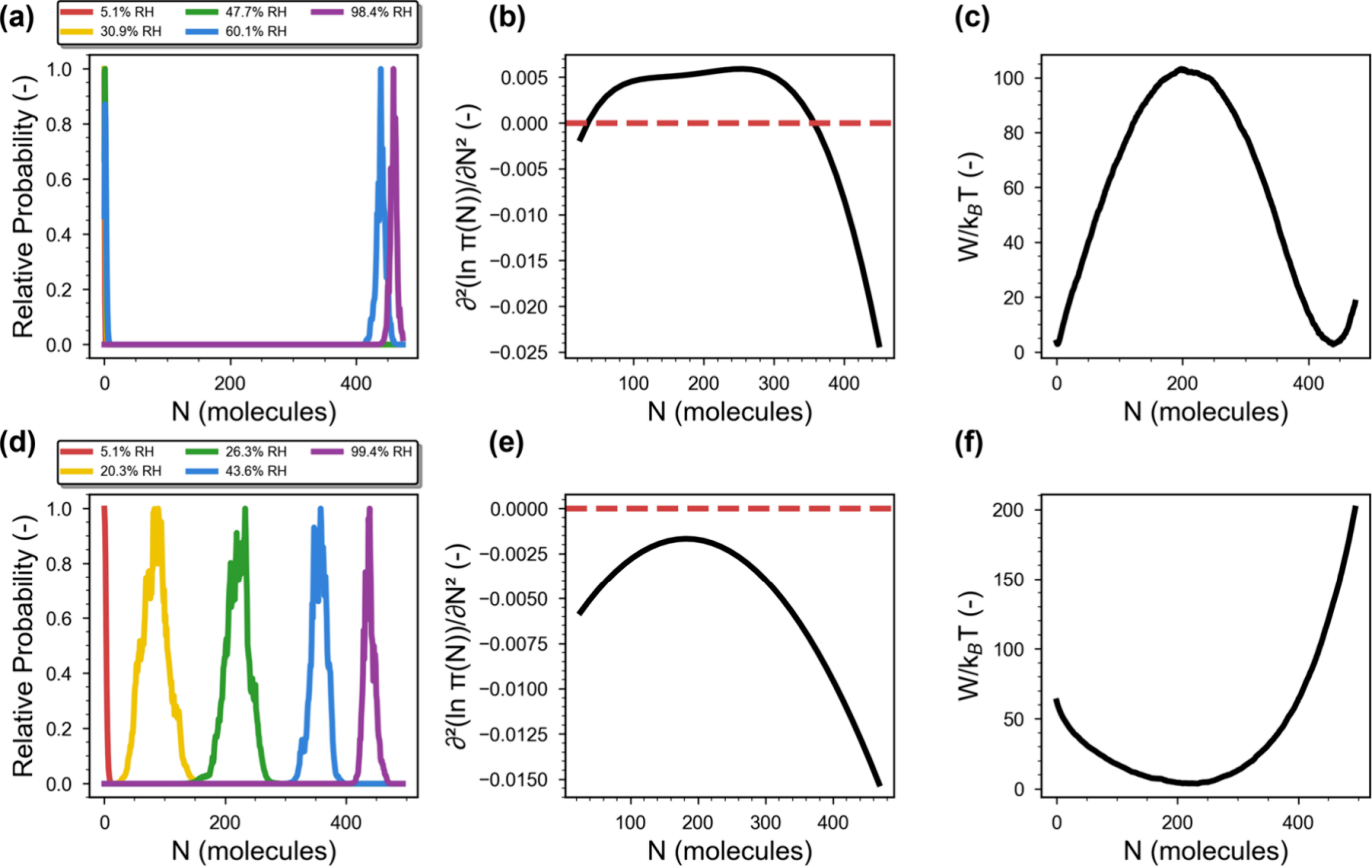
MPD, thermodynamic stability, and grand potential free energy profiles of S1­(I) (a–c, using MOF cg900153m_si_001 as an example structure) and S2­(I) (d–f, using MOF YUBQET as an example structure) isotherms. Both the profiles of (b, e) $\frac{\partial^{2}\ln\Pi }{\partial N^{2}}$ and (c, f) grand potential are evaluated at their step pressures. For the latter, results over the full RH range can be found in SI Section 2.4.

It is well recognized that the fundamental nature of phase transition is governed by the critical temperature *T*_c_ of the system. A system may experience a first-order phase transition at a temperature below *T*_c_, whereas only a single-phase exists above *T*_c_.^52^ As all isotherms presented above are at 298 K, this implies that water in MOFs with bimodal isotherms may have a *T*_c_ above 298 K and vice versa for those unimodal ones. In other words, bimodal isotherms may shift to unimodal, namely, from one that involves a first-order phase transition to a single-phase behavior, at higher temperatures. To test this, the adsorption isotherms of bimodal MOF (ACAJIZ) at 298 K are also computed at elevated temperatures of 318 and 338 K. Indeed, Figure 5a shows that its isotherms progressively shift from bimodal to unimodal profiles. This is also reflected in the pronounced decrease in the free energy barrier as depicted in Figure 5b — from approximately 8 *k*_B_ *T*, 2 *k*_B_ *T,* to no barrier.

**Figure 5:**
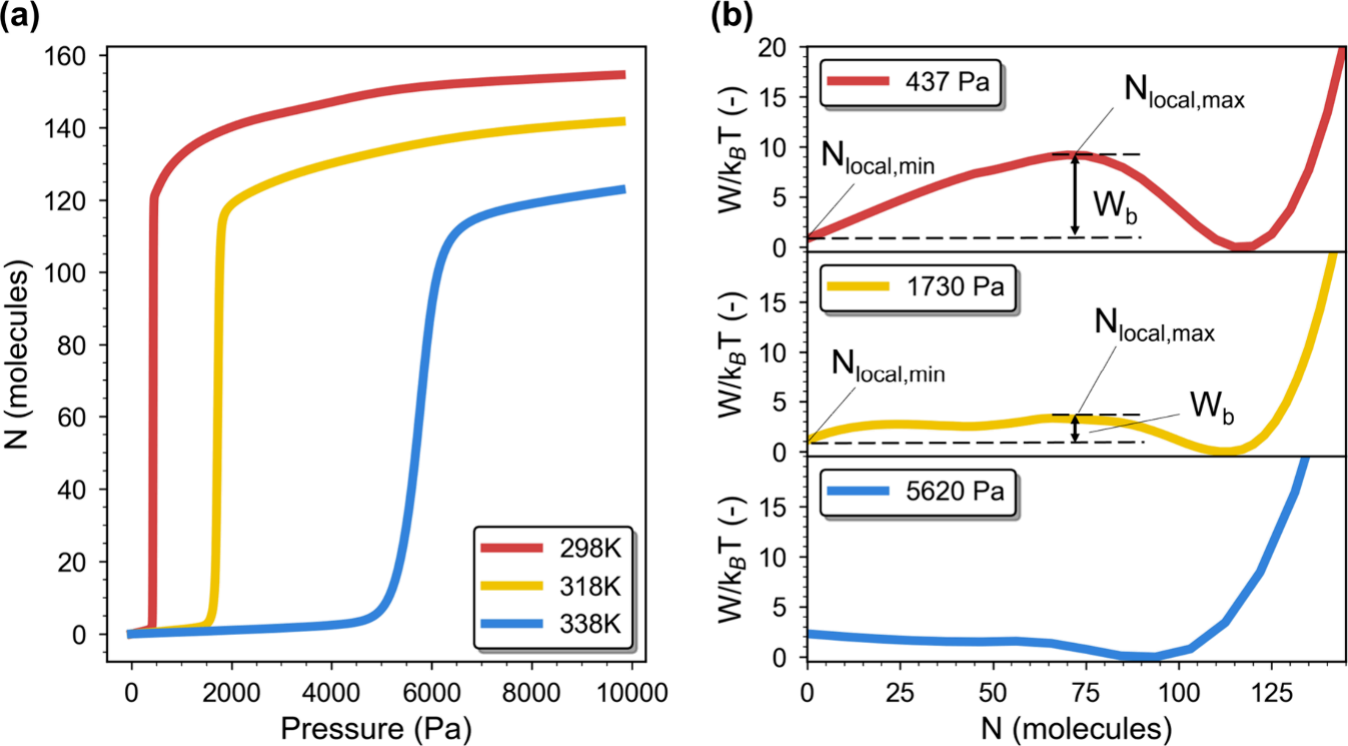
Temperature-dependent water adsorption behavior and free energy profiles of the bimodal MOF (ACAJIZ). (a) Water adsorption isotherms computed at different temperatures of 298, 318, and 338 K. (b) Their corresponding grand potential free energy (*W*) profiles. *W*_b_ is the free-energy barrier between the metastable state and the stable state. *N*_local,min_ is the number of particles at the local minimum of the metastable basin, and *N*_local,max_ is the number of particles at the local maximum of *W*. The minimum *W* of each profile has been shifted to zero for clarity.

### 3.2 Atomic Insights into Adsorption Configurations

With the difference in phase behavior being recognized, it is crucial to also understand how they influence the microscopic nature of adsorption. While water condensation in MOFs is typically considered to be associated with cluster formation,^53–56^ atomic insights into the adsorption configurations of water and distinctions between bimodal and unimodal MOFs remain missing. To this end, this study explores the adsorption configurations in two MOFs with nearly identical largest cavity diameter (LCD) and HoA but distinct phase behaviors: RUGXOI01 (bimodal; LCD = 6.52 Å; HoA = 39.83 kJ/mol) and IWOKUC (unimodal; LCD = 6.56 Å; HoA = 39.51 kJ/mol). As shown in Figure 6, both systems feature sparsely adsorbed water configurations in the early-stage isotherms, serving as nucleation “seeds”. Consistent with the abovementioned prevailing view, these seeds are believed to attract additional molecules and initiate aggregation within the pores.^56,57^ Interestingly, their subsequent adsorption mechanisms are found to diverge markedly. In bimodal RUGXOI01, additional water molecules are still adsorbed in a spatially dispersed and relatively uniform manner, maintaining a gas-like distribution prior to condensation. These molecules gradually interconnect to form extended water clusters, which in turn trigger sudden condensation upon reaching the step pressure. Distinctly, IWOKUC exhibits a continuous and progressive increase in water density. Rather than adsorbing in isolation, additional water molecules accumulate in close proximity to existing ones, resulting in a more localized growth pattern. This behavior is indicative of a continuous transition reminiscent of liquid-like growth and is more in line with the traditional “seeding” mechanism. Similar behaviors are also observed in other MOFs within our data set (SI Section 3.2). These mechanistic differences are further supported by the distinctions in the O–O radial distribution functions (RDFs) of water (SI Section 3.3). As shown in Figure S10, for bimodal MOF RUGXOI01, its RDF shows a sparsely adsorbed water molecules at RH values before condensation, while an RDF profile similarly resembling a typical liquid water emerges after the step pressure. The results clearly indicate a transition from a vapor-like to a liquid-like phase. In contrast, the unimodal MOF IWOKUC distinctly exhibits a gradual decrease in RDF peak intensity with increasing RH, suggesting that water clusters continuously expand as RH increases and finally form a liquid-like phase. These observations are consistent with the density profiles and again reinforcing the proposed mechanistic distinctions.

**Figure 6:**
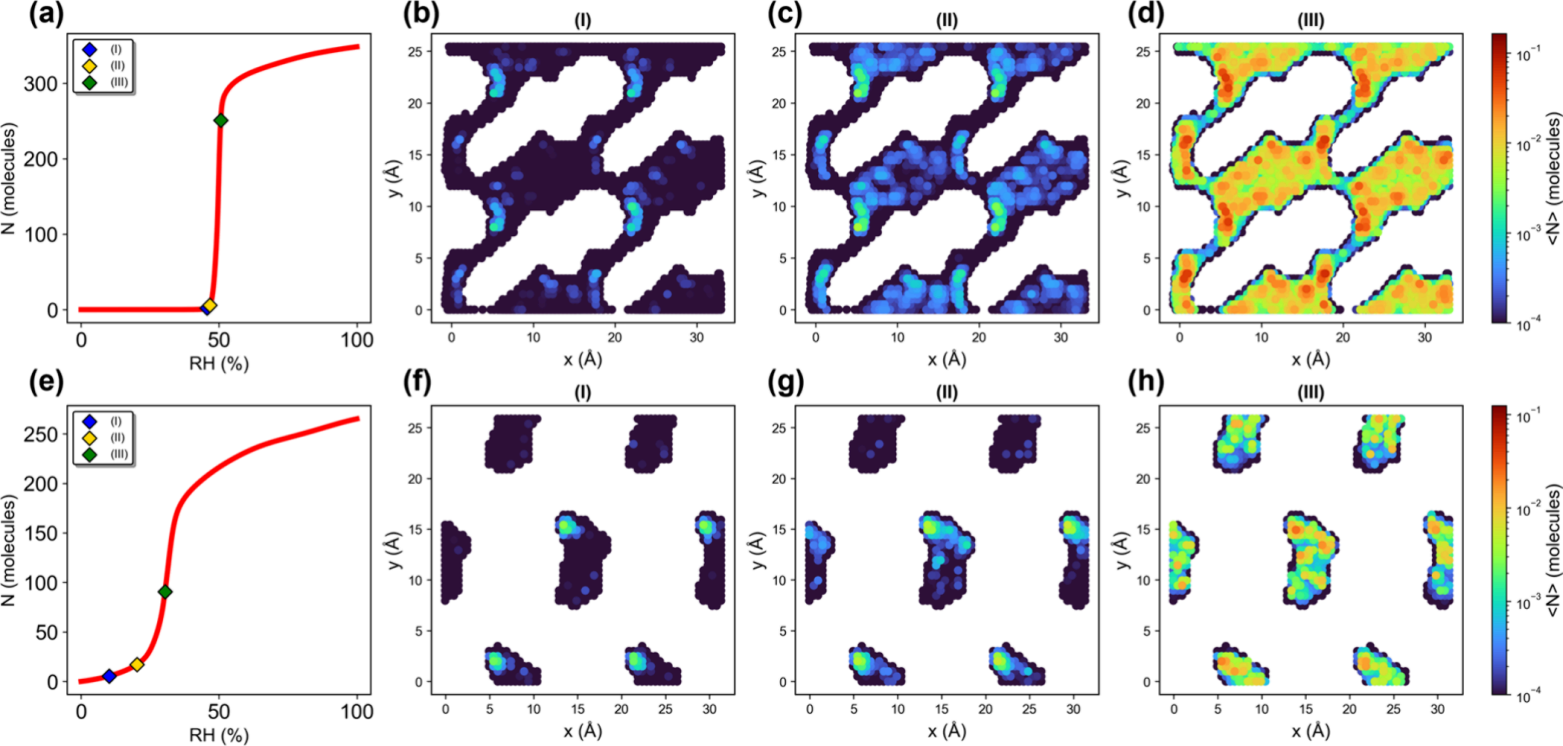
Adsorption isotherms and cross-sectional water adsorption density maps of two S-shaped MOFs: (a–d) bimodal MOF (RUGXOI01) at the *z*-range of 1 to 2.95 Å and (e–h) unimodal MOF (IWOKUC) at the *z*-range of 3 to 3.5 Å. (b–d) and (f–g) show the density maps corresponding to the labeled pressures (I–III) in (a) and (e), respectively. Additional slices for the cross-sectional water adsorption density maps can be seen in SI Section 3.2.

The resulting hydrogen-bonding network is also significantly influenced by the nature of the phase transition. In this study, the number of hydrogen bonds between water molecules adsorbed in MOFs is evaluated at the saturation pressure, with computational details provided in SI Section 4. The hydrogen-bonding networks within bimodal MOFs are found to be more bulk-like than those in unimodal structures (Figure 7). Specifically, bimodal MOFs display a hydrogen bond number that is closer to that of the bulk phase.^58,59^ Moreover, bimodal MOFs have a higher occurrence of double-donor hydrogen bond motifs—a signature characteristic of bulk liquid water.^60^ The supercritical-like adsorption states in unimodal MOFs, by contrast, lead to more disordered hydrogen-bonding states, characterized by a higher proportion of nondonor configurations and a reduced presence of double-donor patterns.

**Figure 7:**
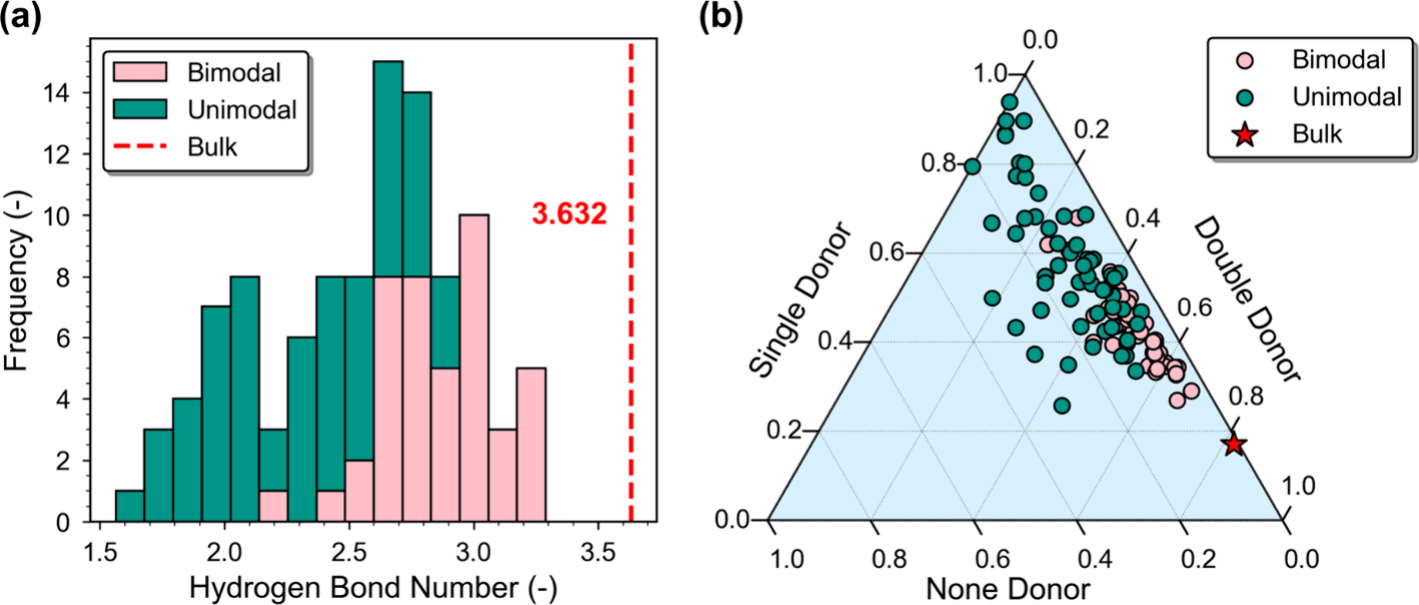
Hydrogen-bonding characteristics of water in bimodal and unimodal MOFs. (a) Distribution of hydrogen bond numbers. (b) Ternary plot showing the distribution of different hydrogen-bonding configurations—double-donor, single-donor, and none-donor.

The results presented above also lead to an interesting discussion on the nature of the phase transition. Although some prior studies and diffraction-based analyses found that water clusters in MOFs show lower mobility than liquid water^61^ or exhibit solid-like configuration,^62^ our results do not support a vapor-to-solid phase transition. Specifically, the above-presented RDF as well as the average number of hydrogen bonds per water molecule (also in previous literature^58,63^ ) both suggest a formation of a liquid-like phase. A direct vapor-to-solid transition might also be thermodynamically implausible under 298 K, as the vapor–solid transition for bulk water occurs below 611.7 Pa and 0.01 °C.^64^

### 3.3 Structure–Property Relationships

Thus, far, our results have clearly revealed distinct types of phase transition, giving rise to differences in adsorption mechanisms and hydrogen-bonding networks. To shed light on the structural origin of these adsorption characteristics, structural–property relationships are further investigated, i.e., how physical and chemical features of MOFs influence the adsorption behavior. This section begins by examining geometric factors, followed by chemical characteristics.

#### Geometric Factors

To elucidate how geometric confinement is associated with adsorption characteristics, LCD, a value that quantifies the size of the largest pore in the structure, is chosen as the representative metric. Figure 8a shows that there exists a correlation between adsorption type and pore size, with 6 Å serving as a clear transition point. Unimodal MOFs are prevalent at LCDs < 6 Å, while bimodal MOFs dominate at larger values. This in fact appears reasonable. As discussed above, the *T*_c_ value for the adsorbed water phase plays a crucial role in determining its phase transition characteristics. Previous studies have also demonstrated that the value of *T*_c_ decreases significantly under strong confinement in various models and real-world materials.^65–75^ Smaller pore lowers the *T*_c_, thereby suppressing phase separation and favoring unimodal adsorption. Motivated by these, vapor–liquid equilibrium (VLE) phase diagrams are also computed using Monte Carlo simulations for water adsorbed in two bimodal MOFs with different LCDs: ACAJIZ (LCD = 6.18 Å) and ic502643m_si_008 (LCD = 9.64 Å). While the VLE of water adsorbed in MOFs can also be probed from their MPD, computing MPD under different temperatures using NVT+W will involve large computational costs. Note that obtaining MPD at temperatures other than the reference one through reweighting involves uncertainties and thus may not be utilized. To this end, this study opts for employing an approach adopted by Smit and co-workers^68^ to simulate a large structural domain under a canonical ensemble and observe VLE directly. More details can be found in SI Section 5. As shown in Figure 8b, the *T*_c_ of confined water in these two MOFs are estimated to be 333.06 and 391.97 K, respectively. Both are substantially lower than the bulk value of 628 K,^76^ and importantly, these results confirm that more confined pores indeed result in reduced *T*_c_. From the hydrogen-bonding perspective, also as previously mentioned, bimodal MOFs tend to exhibit a more bulk-like hydrogen-bonding environment compared to their unimodal counterparts. This behavior is again consistent with their generally greater pore sizes, which offer greater orientational freedom and enable the formation of more extensive hydrogen bond networks. In contrast, the smaller and more confined pores of unimodal MOFs restrict molecular rearrangement and hinder hydrogen bond formation, leading to a more disordered, supercritical state.

**Figure 8:**
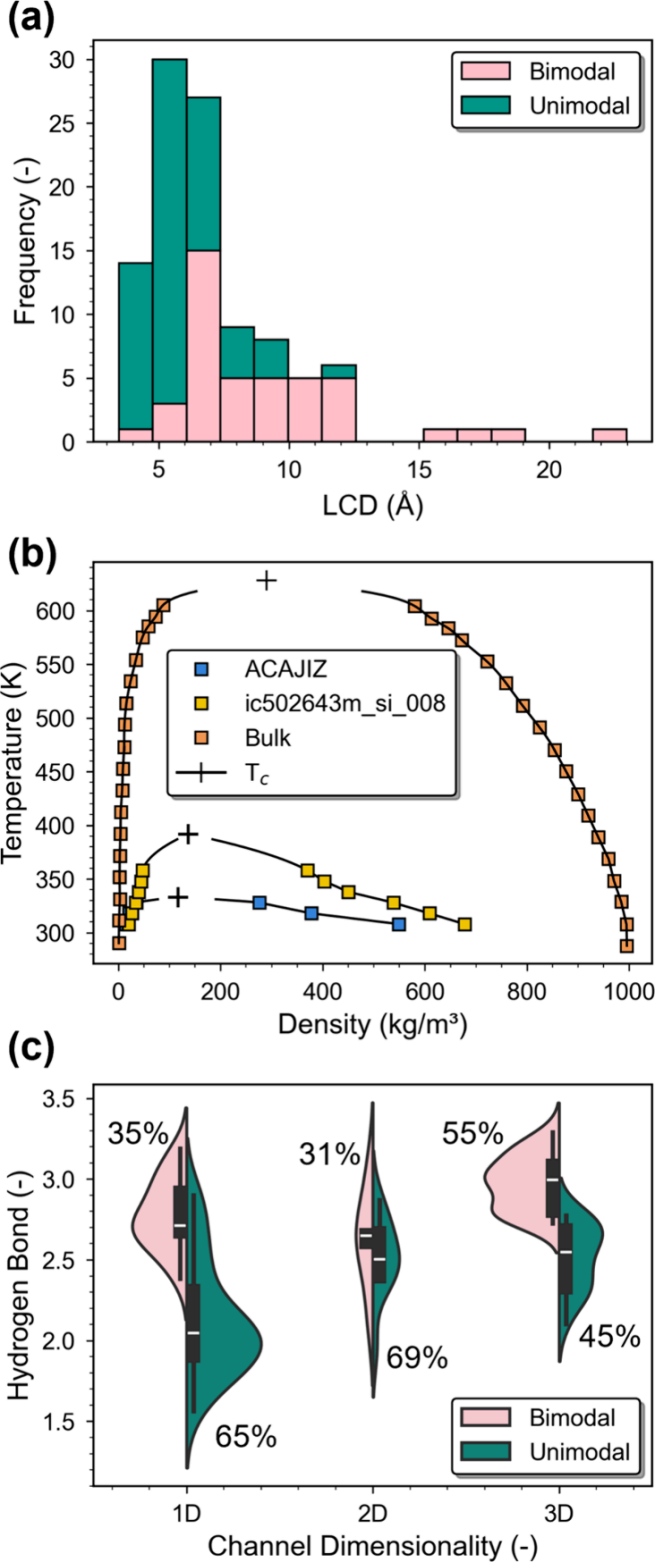
(a) Distribution of bimodal and unimodal MOFs as a function of LCD. (b) Vapor–liquid equilibrium of water adsorbed in ACAJIZ (LCD = 6.18 Å) and ic502643m_si_008 (LCD = 9.64 Å) as well as that of bulk phase (i.e., numerical data are adopted from ref 76). (c) Violin plots of hydrogen bond numbers categorized by channel dimensionality and adsorption types. The width of each violin represents the relative frequency.

Though a trend has been observed, the distributions of bimodal and unimodal MOFs with respect to their pore size still exhibit substantial overlaps. This indicates that MOFs possessing similar pore sizes can give rise to markedly different adsorption behaviors and hydrogen-bonding characteristics. Clearly, as would be expected, factors beyond pore size, such as the spatial arrangement and accessibility of pores, also influence water adsorption characteristics. To further probe this complexity, the influence of channel dimensionality on phase behavior is examined. Herein, the channel dimensionality of the studied MOFs is determined using the Zeo++ package^33,34^ through inspecting the connectivity of accessible voids. Interestingly, Figure 8c suggests that unimodal MOFs are more predominantly associated with 1D channels, with a unimodal-to-bimodal ratio of approximately 65:35. By contrast, bimodal MOFs are more frequently found in 3D channels, with a ratio to be 45:55. That is, MOFs of higher-dimensional channels are more likely to demonstrate bimodal behaviors, possibly owing to their more connected pore topology to better support bulk-like hydrogen bond networks, and vice versa for unimodal behaviors. Note, though, that there is still a quite non-negligible fraction of MOFs with one-dimensional channels that demonstrate bimodal isotherms. These findings highlight that no single structural descriptor, such as pore size or channel dimensionality, fully accounts for the observed complex behavior. Future studies could conduct more comprehensive geometric analyses, such as a more comprehensive quantification of pore connectivity, incorporated with machine learning approaches.

#### Chemical Factors

Aside from geometric confinement, surface functional groups are anticipated to also significantly influence water adsorption behavior. Specifically, this section examines how MOF-water affinity, as reflected by the HoA determined under infinite dilution and the nature of functional groups, affects adsorption characteristics. Figure 9a shows that S-shaped MOFs predominantly exhibit lower HoA, whereas higher HoA, indicative of greater hydrophilicity, is more common to lead to Langmuir-like (i.e., N–S­(I)) isotherms.^47^ This suggests that excessive hydrophilicity may hinder the formation of S-shaped isotherms. Figure 9b further reinforces this trend; MOFs possessing −OH, −NH, and −NH_2_ groups, three well-known hydrophilic groups as examples that are anticipated to lead to stronger interactions with water (i.e., higher HoA values), are not likely to demonstrate S-shaped characteristics. In addition, it is also interestingly observed from Figure 9a that HoA distributions between bimodal and unimodal MOFs are similar, implying that the phase separation of water in MOFs is not strongly governed by framework–water interaction strength. This aligns with the observation that condensation is primarily driven by water–water interactions,^77–79^ whereas HoA are more dominant in the early adsorption stage. This interpretation is also supported by the observation, as shown in Figure 9c, that the subtype (I) of S-shaped isotherms is predominantly associated with low HoA values (∼40 kJ/mol), whereas subtype (II) is more frequently linked to higher HoA values.

**Figure 9:**
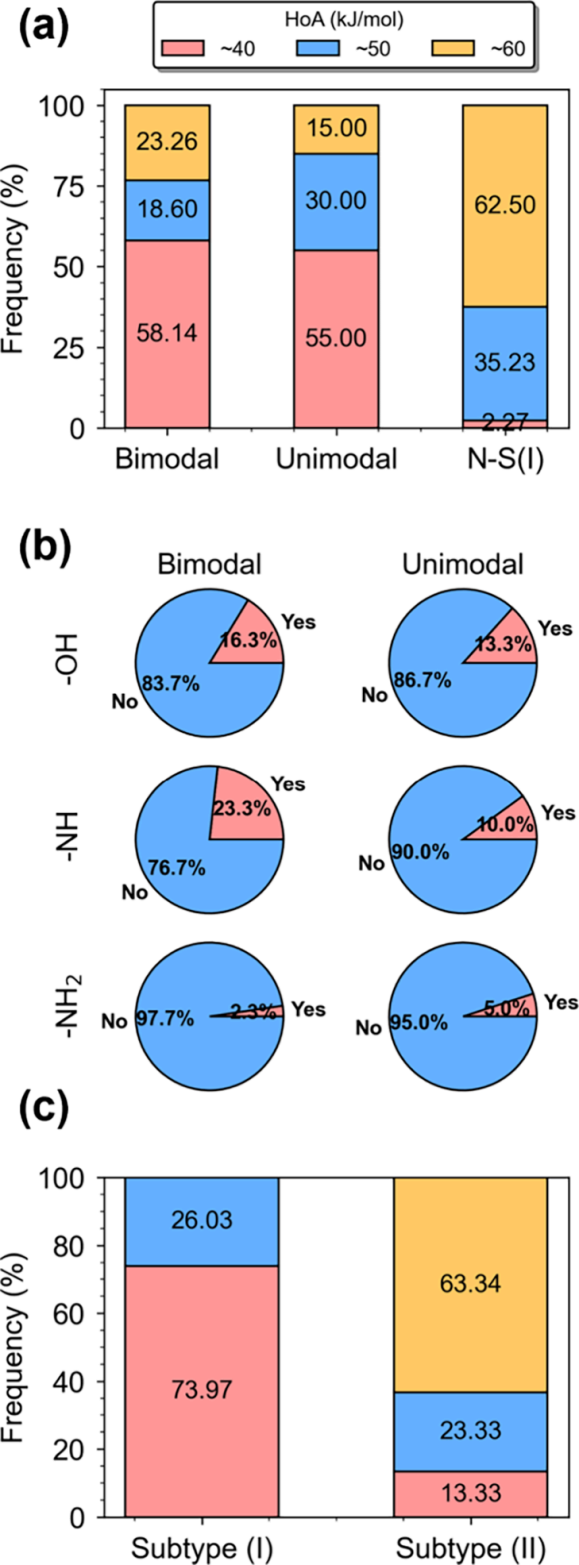
Relationship between chemical properties and water adsorption behavior in MOFs. (a) Distribution of HoA in bimodal, unimodal, and N–S­(I) MOFs. (b) Percentage of MOFs possessing hydrophilic functional groups (i.e., −OH, −NH, and −NH_2_) that demonstrate bimodal or unimodal adsorption characteristics. For instance, 16.3 and 13.3% of MOFs with hydroxyl groups show bimodal and unimodal behaviors, respectively. (c) HoA distribution across S-shaped subtypes.

### 3.4 Step Pressure

Aside from demonstrating S-shaped characteristics, to optimize the effectiveness of an adsorbent in harvesting atmospheric water, its step pressure is also a critical factor. Step pressure is defined as the pressure at which the MOF reaches half of its maximum loading in this study. Similar to the above-presented analysis, the relationship between step pressure and three fundamental MOF features (i.e., LCD, Henry’s constant, and HoA) is also analyzed. Figure 10a–c shows that Spearman correlation coefficients, *r*_s_, between step pressure and these properties are found to be 0.14, −0.35, and −0.24. That is, while a more bulk-like pore environment (larger LCD) and stronger framework–water affinity (higher Henry’s constant and HoA) indeed influence the location of the step pressure, the correlations can be deemed rather weak as would be somewhat expected.

**Figure 10:**
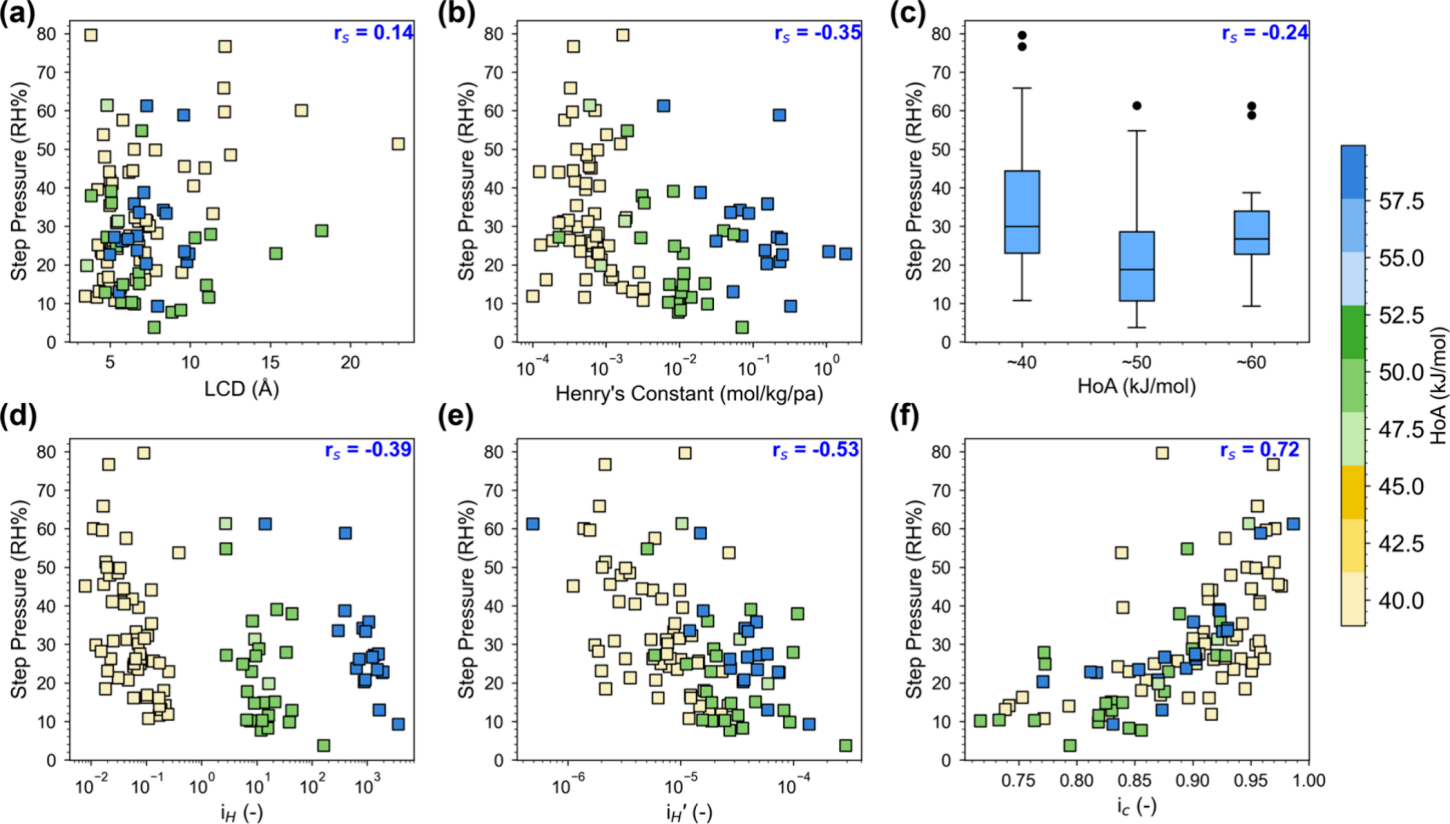
Correlations between step pressure and (a) largest cavity diameter (LCD), (b) Henry’s constant, (c) heat of adsorption (HoA), (d) hydrophilicity index (*i*_H_), (e) simplified hydrophilicity index without the exponential HoA weighting (*i*_H_^′^), and (f) newly developed connectivity index (*i*_c_) for S-shaped MOFs. Additional computational details for *i*_H_ and *i*_c_ are provided in SI Section 6. Note that in (c), the results are grouped into the three representative HoA values (i.e., ∼40, ∼50, and −60 kJ/mol).

To improve upon these basic descriptors, Nguyen et al.^80^ recently introduced a so-called hydrophilicity index (*i*_H_), which integrates both the density and energetic quality of adsorption sites:

$$i_{H}=\frac{N_{ads}}{S_{A}}\exp\left(-\frac{\overset{®}{\Delta H_{ads}}}{RT}\right)$$ 1

where *N*_ads_ is the number of adsorption sites within the unit cell, *S*_A_ is the theoretical surface area, $\overset{®}{\Delta H_{ads}}$ is the average isosteric heat of water adsorption. Herein, adsorption sites are identified as sites with local energy below −4730K (i.e., 9.4 kcal/mol) following the definition in their study^80^ based on the number of energy grids under infinite dilution that meet this threshold. For $\overset{®}{\Delta H_{ads}}$ , its values were originally determined from averaging the isosteric heats calculated via applying the Clausius–Clapeyron equation to experimental isotherms at multiple temperatures over different loadings. However, extending this to a large data set of 225 MOFs would be too computationally intensive. As a more practical and scalable alternative, this study adopts the isosteric heat under infinite dilution as a proxy for $\overset{®}{\Delta H_{ads}}$ for its capability to capture the intrinsic guest–host affinity that governs initial adsorption. While this hydrophilicity index is promising in their data set consisting of 19 structures (*r*_s_ = −0.94, as shown in SI Section 6), it shows limited predictive power in our diverse data set of >200 MOFs (*r*_s_ = −0.39), as shown in Figure 10d. Notably, its limited performance appears to stem, in part, from the exponential weighting term involving $\overset{®}{\Delta H_{ads}}$ : *i*_H_ performs well among materials with similar HoA but fails when comparing materials with a broader range of HoA. This is further confirmed after removing the factor of $\exp\left(-\frac{\overset{®}{\Delta H_{ads}}}{RT}\right)$ . As shown in Figure 10e, the resulting density of adsorption sites, ${i_{H}}^{\prime }=\frac{N_{\mathrm{ads}}}{S_{A}}$ , has a stronger correlation of *r*_s_ = −0.53. This finding aligns with the above discussion that the condensation of water is primarily triggered by the formation of water clusters rather than framework–water interactions.

Thus far, the results suggest that employing the density of adsorption sites alone may be insufficient. As reported from a recent study by some of us,^79^ the existence of continuous adsorption channels (CACs) can largely promote the water–water interactions, indicating that intermolecular connectivity among water molecules is also a critical factor. To capture this dual requirement, we further introduce the “connectivity index (*i*_c_)”, a new descriptor that integrates density, connectivity, and spatial homogeneity per the energy grid under infinite dilution:

$$i_{c}=\frac{\overset{n}{\underset{i=1}{\sum }}\left(2i-n-1\right){D_{ads}}_{i}}{n\overset{n}{\underset{i=1}{\sum }}{D_{ads}}_{i}}$$ 2

$${D_{ads}}_{i}=\frac{{N_{ads}}_{i}}{V_{A}}$$ 3

where *D*_ads_i__ represents the local adsorption site density within a spherical region of 3 Å diameter centered at grid *i*, sorted in ascending order, *N*_ads_i__ is the number of adsorption sites within that local region determined using the same energy threshold (i.e., −4730 K), *V*_A_ is the accessible volume, and *n* is the total number of grids. These quantities are all determined per single unit cell. The 3 Å diameter corresponds to the weakest hydrogen bond length,^81^ ensuring that molecules within this region are potentially connected through hydrogen-bonding interactions. Accordingly, *D*_ads_i__, distinct from the abovementioned *N*_ads_, effectively reflects not only the local density but more importantly also the spatial connectivity of adsorption sites. With *D*_ads_i__, per a classical measure of inequality—the Gini coefficient—the homogeneity of all locally dense, connected regions is further quantified. The resulting *i*_c_ (eq 2) has values ranging from 0 (perfect homogeneity) to 1 (maximum heterogeneity). Notably, as shown in Figure 10f, *i*_c_ achieves an improved correlation with step pressure (*r*_s_ = 0.72). MOFs with lower *i*_c_ values tend to condense water at lower RH, indicating that more uniform adsorption sites facilitate CAC formation at earlier stages. This trend is especially evident for bimodal MOFs, which shows a stronger correlation (*r*_s_ = 0.75) compared to unimodal ones (*r*_s_ = 0.66) (SI Section 6). The better performance of *i*_c_ in bimodal MOFs again supports the mechanism discussed above for bimodal isotherms featuring more uniform adsorption.

At this point, it is important to note that the value of −4730 K as the energy threshold for identifying favorable adsorption sites may be deemed as an arbitrary choice. Alternative thresholds are tested to evaluate the sensitivity of *i*_c_. Specifically, the −4500 K threshold adopted by Xu et al.^79^ is also used, along with a variety of scaled values of this value (i.e., −3600, −4050, and −4950 K). As shown in SI Section 6, *r*_s_ increases from 0.58 at −3600 K to 0.67 at −4050 K, peaks at 0.73 with −4500 K, and slightly declines to 0.68 at −4950 K. These results indicate that while *i*_c_ is somewhat sensitive to the energy threshold, its predictive strength remains consistently high, demonstrating robustness across reasonable threshold values. However, while the newly proposed index is found to better correlate with the step pressure with a decent *r*_s_ of more than 0.7, its prediction capability can still be deemed rather restricted. This is again attributed to the highly complex nature of water adsorption. Nonetheless, the results evidently suggest that the homogeneity and connectivity of water in MOFs are critical to their adsorption behaviors. For this, machine learning approaches, particularly convolutional neutron networks (CNNs), may hold promises to predict the step pressure. CNN models, as shown in several seminal studies reported in the literature, have also been successfully developed to predict the adsorption of methane and carbon dioxide with superior accuracy.^82–84^

## 4 Conclusions

This study investigates water adsorption in >200 diverse MOFs using flat histogram Monte Carlo simulations. Diverse characteristics, including non-S-shaped and S-shaped isotherms, are observed. S-shaped isotherms are further found to be associated with two distinct phase behaviors. Isotherms with a sharp uptake increase exhibit “bimodal” MPDs, indicative of first-order phase transitions, whereas those with relatively moderately increased uptake correspond to “unimodal” MPDs with a single, continuous phase. These phase distinctions lead to significantly different adsorption mechanisms and hydrogen-bonding environments: bimodal MOFs show spatially dispersed adsorption and more liquid-like hydrogen-bonding networks, while unimodal MOFs exhibit cluster-based growth and supercritical-like environments. The occurrence of these transitions is governed by the critical temperature of confined water, as evidenced by the shift from bimodal to unimodal isotherms at elevated temperatures. Structure–property relationships are also analyzed in this study. Low heat of adsorption under infinite dilution and a minimal presence of hydrophilic functional groups enables the formation of “S-shape”. Large pores elevate the critical temperature of confined water, thereby increasing the likelihood of phase separation. High channel dimensionality also facilitates the development of bulk-like hydrogen-bonding networks, further supporting the emergence of distinct phase transitions. Lastly, factors governing the step pressure of water condensation are explored, and a new, robust connectivity index is developed. MOFs with more uniform adsorption environments tend to exhibit lower step pressures. Based on these findings, optimal MOFs for water harvesting should typically exhibit moderate hydrophilicity, relatively larger pore sizes, higher channel dimensionality, and lower connectivity index. Altogether, this work offers a comprehensive understanding toward water adsorption in MOFs. The outcomes of this work are anticipated to greatly facilitate the development of next-generation nanoporous adsorbents for efficient atmospheric water harvesting.

## Supplementary Material





## Notes

Additional details include the theoretical framework of NVT+W simulations, force field parameters, isotherm convergence criterion, and computational details of thermodynamic properties, adsorption site analysis, hydrogen bond networks, VLE diagrams, hydrophilicity index, and connectivity index (PDF)
The results of all studied 225 MOFs, including their HoA, isotherm types, LCD, channel dimensionality, number and ratio of hydrogen bonds, presence of hydrophilic functional groups (−OH, −NH, and -NH_2_), and values of the hydrophilicity index (*i*_H_ and *i*_H_^′^) and the connectivity index *i*_c_ under various energy thresholds (−3600, −4050, −4500, −4730, and −4950 K) (XLSX)
