# Supplementary material for: Water Adsorption in Metal–Organic Frameworks: Characteristics, Mechanisms, and Structure–Property Relationships
Source: J Am Chem Soc. 2025 Sep 10;147(38):34791–803. doi: 10.1021/jacs.5c10686 (PMC12464999; doi:10.1021/jacs.5c10686)
Supplement: Supplementary file 1 [file ja5c10686_si_001.pdf]

Supporting Information

Water Adsorption in Metal-Organic Frameworks: Characteristics, Mechanisms, and Structure-Property Relationships

Shiue-Min Shih[a] and Li-Chiang Lin[a,b,*]

[a]Department of Chemical Engineering, National Taiwan University, Taipei 106319, Taiwan

[b]William G. Lowrie Department of Chemical and Biomolecular Engineering, The Ohio State University, Columbus, Ohio 43210, United States

[*]Email: lclin@ntu.edu.tw

Table of Contents

1. NVT+W Simulations 1

 1.1. Theoretical Framework 1

 1.2. MPD Reweighting 3

 1.3. Force Field Parameters 4

 1.4. Convergence Criteria 7

2. Water Adsorption Characteristics 8

 2.1. Interaction Energies of Non-S-shaped (N-S) Isotherms 8

 2.2. Macrostate Probability Distribution 9

 2.3. Thermodynamic Analysis – Stability Limit and Grand Potential Free Energy ... 11

 2.4. Grand Potential Free Energy Profile 13

3. Water Adsorption Mechanism 14

 3.1. Adsorption Site Analysis 14

 3.2. Density Maps 16

 3.3. Radial Distribution Functions (RDFs) of Water Adsorbed in MOFs 20

4. Hydrogen Bond Networks 21

5. Vapor-Liquid Equilibrium (VLE) and Critical Properties 22

 5.1. Computational Details 22

 5.2. Critical Properties 27

6. Step Pressure 28

7. References 31

1. NVT+W Simulations

1.1. Theoretical Framework

The NVT+W method[1, 2] is a kind of flat histogram Monte Carlo simulation. This approach essentially employs Transition Matrix Monte Carlo (TMMC) simulations with a bin size of 1[3, 4]. The core concept of TMMC introduces a collection matrix C (C-matrix) to obtain the macrostate probability distribution (MPD), $\Pi(N;\mu,V,T)$, which can then be employed to compute the average loading. Simulations are conducted across all accessible macrostates under a fixed chemical potential μ, volume V and temperature T, i.e., from $N=0$ to $N=N_{max}$, where N_{max} is the saturation loading of the adsorbent. Unlike grand canonical Monte Carlo (GCMC), this method does not execute real swap moves but only simulates their insertion or deletion attempts without altering the number of particles in the system.

In each NVT+W simulation, millions of translation, rotation, reinsertion, and Widom insertion/deletion moves are performed. The C-matrix accumulates the unbiased acceptance probabilities for Widom insertion and deletion moves per grand canonical ensemble:

Widom insertion

$$C(N \to N+1) = C(N \to N+1) + \alpha(\Gamma_r^N \to \Gamma_r^{N+1}) \quad \text{(S1)}$$

$$C(N \to N) = C(N \to N) + 1 - \alpha(\Gamma_r^N \to \Gamma_r^{N+1}) \quad \text{(S2)}$$

$$\alpha(\Gamma_r^N \to \Gamma_r^{N+1}) = \min\,[1, \frac{\beta V \phi P}{N+1} e^{-\beta(U(\Gamma_r^{N+1})-U(\Gamma_r^N))}] \quad \text{(S3)}$$

Widom deletion

$$C(N \to N-1) = C(N \to N-1) + \alpha(\Gamma_r^N \to \Gamma_r^{N-1}) \quad \text{(S4)}$$

$$C(N \to N) = C(N \to N) + 1 - \alpha(\Gamma_r^N \to \Gamma_r^{N-1}) \quad \text{(S5)}$$

$$\alpha(\Gamma_r^N \to \Gamma_r^{N-1}) = \min\,[1, \frac{N}{\beta V \phi P} e^{-\beta(U(\Gamma_r^{N-1})-U(\Gamma_r^N))}] \quad \text{(S6)}$$

where $C(N \to N \pm 1)$ records the cumulative acceptance probabilities for Widom insertions and deletions, $C(N \to N)$ accumulates the rejection probabilities for both types of Widom moves, α is the acceptance probability, Γ_r^N, Γ_r^{N+1}, and Γ_r^{N-1} respectively represent microstates in macrostate N, $N+1$, and $N-1$, β is the inverse temperature ($\beta = 1/k_B T$, where k_B is the Boltzmann constant), and ϕ is the fugacity coefficient. Note that $C(N \to N-1) = 0$ for $N = 0$. Subsequently, the macrostate transition probability matrix P can be computed from the C-matrix:

$$P(N \rightarrow N + \Delta\mathrm{N}) = \frac{C(N \rightarrow N + \Delta\mathrm{N})}{\sum_{\Delta\mathrm{N}} C(N \rightarrow N + \Delta\mathrm{N})} \tag{S7}$$

, where $P(N \rightarrow N + \Delta\mathrm{N})$ is the probability of the system moving from macrostate N to $N + \Delta\mathrm{N}$, with $\Delta N = -1, 0, +1.$ With detailed balance, relative probabilities across all macrostates can then be determined as shown in Eq. (S8):

$$\Pi(N; \mu, V, T) P(N \rightarrow N + 1) = \Pi(N + 1; \mu, V, T) P(N + 1 \rightarrow N) \tag{S8}$$

Once the macrostate probabilities are normalized, (i.e., $\sum_{N=0}^{N_{max}} \Pi(N) = 1$), one can obtain the MPD. The average loading is then computed as:

$$< N >= \frac{\sum_{N=0}^{N_{max}} N \cdot \Pi(N; \mu, V, T)}{\sum_{N=0}^{N_{max}} \Pi(N; \mu, V, T)} \tag{S9}$$

Note though that calculating the average loading does not require normalized macrostate probabilities. It can be determined by expressing all $\Pi(N; \mu, V, T)$ values relative to a reference macrostate, such as $\Pi(N = 0)$.

1.2. MPD Reweighting

An important advantage of the flat histogram method is that the MPD at a particular μ, V, T can be reweighted to MPD at other chemical potential μ'. For more details, readers are referred to the study of Datar et al[5]. The probability distribution of macrostate *N*, can be expressed by:

$$\Pi(N; \mu, V, T) = \frac{\exp(\beta\mu N)\, Q(N, V, T)}{\Xi(\mu, V, T)} \tag{S10}$$

where $Q(N, V, T)$ and $\Xi(\mu, V, T)$ represent the canonical and grand canonical partition function, respectively. Similarly, the probability distribution of macrostate *N* at μ' under the same temperature, $\Pi(N; \mu', V, T)$, is:

$$\Pi(N; \mu', V, T) = \frac{\exp(\beta\mu' N)\, Q(N, V, T)}{\Xi(\mu', V, T)} \tag{S11}$$

After taking the natural logarithm on both sides of Eq. (S10) and Eq. (S11), and using $\Pi(0; \mu, V, T)$ and $\Pi(0; \mu', V, T)$ as reference states, Eq. (S12) can then be obtained by subtracting the two equations.

$$\ln \frac{\Pi(N; \mu', V, T)}{\Pi(0; \mu', V, T)} = \ln \frac{\Pi(N; \mu, V, T)}{\Pi(0; \mu, V, T)} + \beta(\mu' - \mu)N \tag{S12}$$

This equation allows the average loading to be determined at any desired pressure, yielding a full isotherm with infinite resolution. It is important to note that the MPD can also be reweighted to different temperatures. For this, readers are referred to the study of Witman et al.[1]

1.3. Force Field Parameters

The Lennard-Jones (L-J) parameters of framework atoms (**Table S1**) are sourced from DREIDING[6], with those unavailable to be instead adopted from UFF[7]. Water molecules are modeled using the TIP4P-EW model[8] (**Table S2**).

Table S1. L-J parameters for framework atoms.

**Atom Type**	ε/k_B **(K)**	**σ (Å)**
Ag	18.12	2.80
Al	156.00	3.91
As	155.47	3.77
Au	19.62	2.93
B	47.81	3.58
Ba	183.15	3.30
Be	42.77	2.45
Bi	260.63	3.89
Br	186.19	3.52
C	47.86	3.47
Ca	119.75	3.03
Cd	114.73	2.54
Ce	6.54	3.17
Cl	142.56	3.52
Co	7.05	2.56
Cr	7.55	2.69
Cs	22.64	4.02
Cu	2.52	3.11

Dy	3.52	3.05
F	36.48	3.09
Fe	27.69	4.04
Ga	208.84	3.90
Gd	4.53	3.00
Ge	190.69	3.81
H	7.65	2.85
I	256.77	3.70
K	17.61	3.40
La	8.55	3.14
Li	12.58	2.18
Mg	55.86	2.69
Mn	6.54	2.64
Mo	28.18	2.72
N	38.95	3.26
Na	251.70	2.80
Ni	7.55	2.52
O	48.18	3.03
P	161.03	3.70
Pb	333.59	3.83
Pd	24.15	2.58
Pt	40.25	2.45
Rb	20.13	3.67
Ru	28.18	2.64

S	173.11	3.59
Sb	225.95	3.94
Sc	9.56	2.94
Se	146.42	3.75
Si	156.08	3.80
Sn	285.28	3.91
Sr	118.24	3.24
Ti	8.55	2.83
V	8.05	2.80
W	33.71	2.73
Y	36.23	2.98
Zn	27.68	4.05

Table S2. L-J parameters and atomic charges for water molecules.

**Atom Type**	$\boldsymbol{\varepsilon/k_B}$ **(K)**	**σ (Å)**	**q (-)**
O	81.92	3.16	0.000
H	0.00	0.00	0.524
M	0.00	0.00	-1.048

1.4. Convergence Criteria

All simulations are conducted until a proper convergence is achieved – the resulting isotherm remains unchanged with a doubled number of cycles. With this criterion, most simulations are conducted with 20,000 initialization cycles followed by 800,000 production cycles. As illustrated in **Figure S1**, 800,000 production cycles are sufficient to achieve converged results. Besides, the macrostate probability of N_{max} under 986 Pa (i.e., P/P_0 =1 per the chosen water model, where P is the pressure and P_0 is the saturation pressure) is also verified to approach zero to ensure that all accessible macrostates are adequately sampled. In other words, the chosen N_{max} can be deemed sufficiently large such that all thermodynamically relevant macrostates are considered.

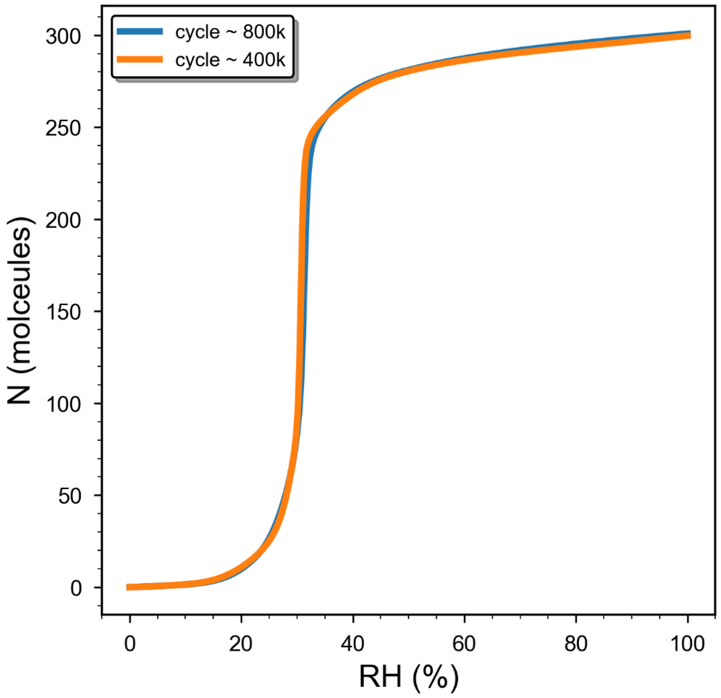

Figure S1. Converged isotherms for MOF DOYHUW. Convergence is defined as the condition where the adsorption isotherm remains unchanged upon doubling the number of cycles in this study. In this example, the isotherm shows no significant changes when the number of cycles is increased from 400,000 to 800,000.

2. Water Adsorption Characteristics

2.1. Interaction Energies of Non-S-shaped (N-S) Isotherms

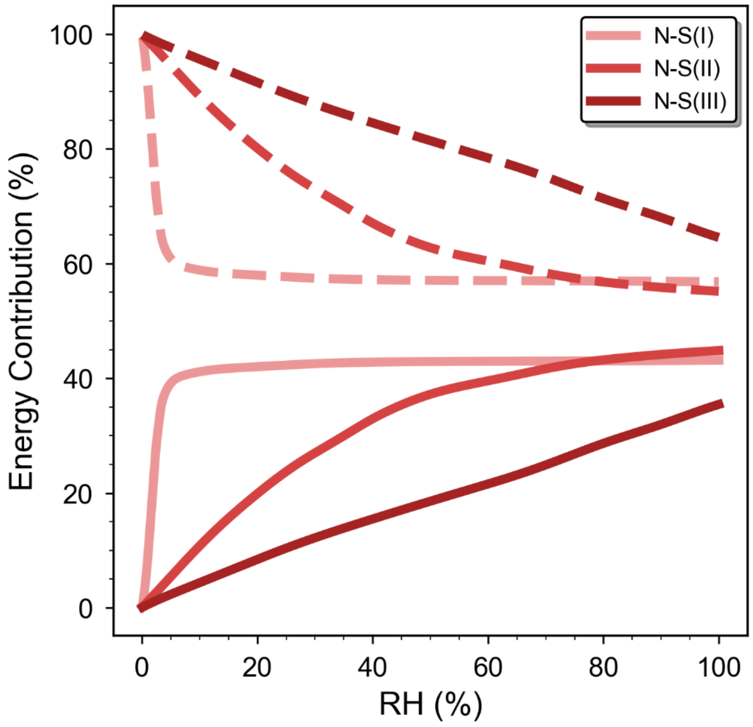

Figure S2. Interaction energy contributions for water in three non-S-shaped (N-S) MOFs: N-S(I), N-S(II), and N-S(III). The MOF–water and water–water interactions are denoted by dashed and solid lines, respectively. As the RH increases, the contribution from MOF–water interactions reduces most rapidly in N-S(I), followed by N-S(II) and then N-S(III). This trend suggests that N-S(I) adsorption is primarily driven by the strong hydrophilicity of the framework, while N-S(II) and N-S(III) increasingly rely on water–water interactions.

2.2. Macrostate Probability Distribution

To understand the thermodynamic origins behind the four distinct types of S-shaped isotherms, this study examines the MPD and grand potential free energy profile of water adsorbed in four representative MOFs: S1(I) – cg900153m_si_001, S1(II) – ALEJIM, S2(I) – YUBQET, and S2(II) – PEWGEF. The corresponding water adsorption isotherms of these materials are shown in **Figure S3**. The MPD results are discussed in this section, while the grand potential free energy analysis is detailed in Section 2.4.

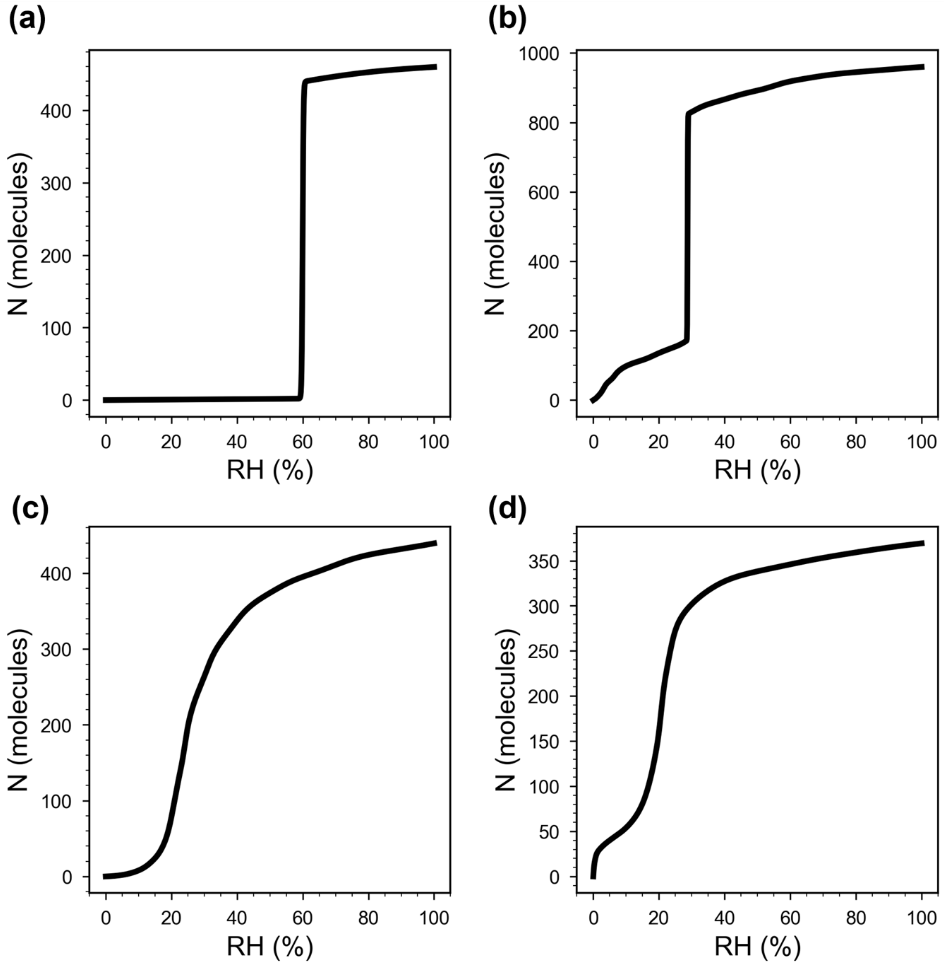

Figure S3. Water adsorption isotherms of the four representative MOFs. **(a)** S1(I): cg900153m_si_001; **(b)** S1(II): ALEJIM; **(c)** S2(I): YUBQET; **(d)** S2(II): PEWGEF.

MPD profiles of the subtype (II) for both S1 and S2 isotherms are shown in **Figure S4**. These subtypes are found to exhibit adsorption behavior consistent with their subtype (I) counterparts: the S1(II) isotherm displays a bimodal MPD, indicative of phase separation, whereas the S2(II) isotherm maintains a unimodal MPD, reflecting a continuous adsorption process.

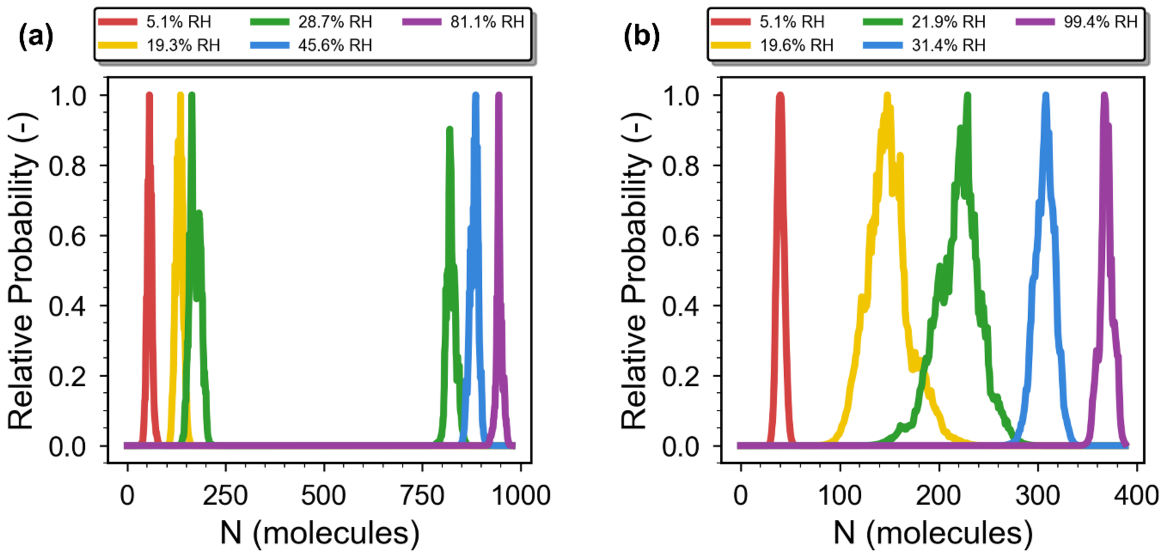

Figure S4. MPD of the subtype (II) of S1 and S2 isotherms: **(a)** S1(II) (MOF ALEJIM) and **(b)** S2(II) (MOF PEWGEF).

2.3. Thermodynamic Analysis – Stability Limit and Grand Potential Free Energy

Understanding the underlying physics behind adsorption isotherms requires clear insights into the phase behavior of the system, which can be also probed from both the thermodynamic stability and grand potential free energy. Remarkably, the MPD also offers a direct and powerful means of probing these properties. This section introduces the methodology for deriving these thermodynamic properties using MPD.

Thermodynamic stability limit: For a stable, single-phase, pure-material system, the second derivative of its Helmholtz free energy *A* with respect to *N*, as shown in Eq. (S13), must be positive for all values of *N* at fixed *V* and *T*.[9]

$$\frac{\partial^2}{\partial N^2}[A(N,V,T)]_{T,V} > 0 \quad \text{(S13)}$$

In contrast, for systems involving first-order (discontinuous) phase transition[10], it has unstable region where the second derivative becomes negative. This quantity, $\frac{\partial^2 A}{\partial N^2}$, can be directly derived from MPD. From statistical mechanics, the probability distribution of macrostate *N*, $\Pi(\mathrm{N};\mu,\mathrm{V},\mathrm{T})$, is expressed as Eq. (S10). Taking the natural logarithms of both sides in Eq. (S10) with rearrangements leads to the following Eq. (S14).

$$ln\, Q(N,V,T) = \ln \Pi(N;\mu,V,T) + \ln \Xi(\mu,V,T) - \beta\mu N \quad \text{(S14)}$$

The Helmholtz free energy A, per its definition, and its second derivative can then be determined as shown in Eq. (S15) and Eq. (S16), respectively.

$$\beta A = -\ln Q(N,V,T) = -\ln \Pi(N;\mu,V,T) - \ln \Xi(\mu,V,T) + \beta\mu N \quad \text{(S15)}$$

$$\frac{\partial^2 A}{\partial N^2} = -k_B T \frac{\partial^2 \ln \Pi(N;\mu,V,T)}{\partial N^2} \quad \text{(S16)}$$

Per both Eq. (S13) and Eq. (S16), for a stable single-phase system, the MPD must satisfy:

$$\left[\frac{\partial^2 \ln \Pi}{\partial N^2}\right]_{T,V} < 0 \quad \text{(S17)}$$

which indicates that the MPD profile is strictly unimodal. By contrast, during phase separation, $\frac{\partial^2 \ln \Pi}{\partial N^2}$ becomes positive between the two spinodal points where the second derivative is zero. This leads to inflection points, corresponding to a bimodal MPD distribution.

Grand potential free energy: the grand potential free energy W, that can be derived as well from MPD (Eq. (S18)), also serves as a key descriptor for characterizing phase behaviors.

$$\beta W(N; \mu V\, T) = -\ln \Pi(N; \mu V T) \tag{S18}$$

This offers an alternative route to identify the relative favorability of different macrostates. $W(N; \mu V\, T)$ for a single-phase system will show a single-well shape, whereas those undergoing phase separation will exhibit a double-well form. For the latter, the higher-energy well corresponds to a metastable state and the lower-energy well represents the **stable** equilibrium phase**.** The free-energy barrier between the two states, $W_b(\mu, V, T)$, can also be quantified by[4]:

$$\beta W_b(\mu, V, T) = -ln \frac{\Pi(N_{local,max};\ \mu, V, T)}{\Pi(N_{local,min};\ \mu, V, T)} \tag{S19}$$

where $N_{local,min}$ is the number of particles at the local minimum of the metastable basin, and $N_{local,max}$ is the number of particles at the local maximum of the free energy. Note that Eq. (S19) can be further applied to determine the nucleation rate[4], informing the kinetic stability of the system. This aspect though is out of the scope of this study.

2.4. Grand Potential Free Energy Profile

In addition to the free energy profile at the step pressure presented in the main text, **Figure S5** shows free energy profiles under the entire RH range to provide a more comprehensive view of the phase transition in bimodal and unimodal MOFs. For the bimodal MOF, a stable free energy minimum appears at N=1 under a 15.2% RH, indicating a vapor-like phase. As RH increases to 29.4%, the minimum persists, while a local plateau emerges at a higher loading, signaling the formation of a metastable liquid-like phase. At 47.7% RH, this plateau deepens into a metastable basin. By 60.1% RH (the step pressure), a distinct double-well profile appears with two comparable minima, indicating vapor–liquid phase coexistence. At 98.4% RH, the system fully transitions to a liquid-like phase, reflected by a single, deep minimum at high loading. These features collectively indicate a first-order phase transition, marked by an abrupt shift from vapor-like to liquid-like states near the step pressure. By contrast, the unimodal MOF displays a qualitatively different profile. Across all RH values, the grand potential remains single-welled, without evidence of metastability or coexistence. This behavior is consistent with a continuous transition, wherein water molecules gradually fill the pore space with increasing RH.

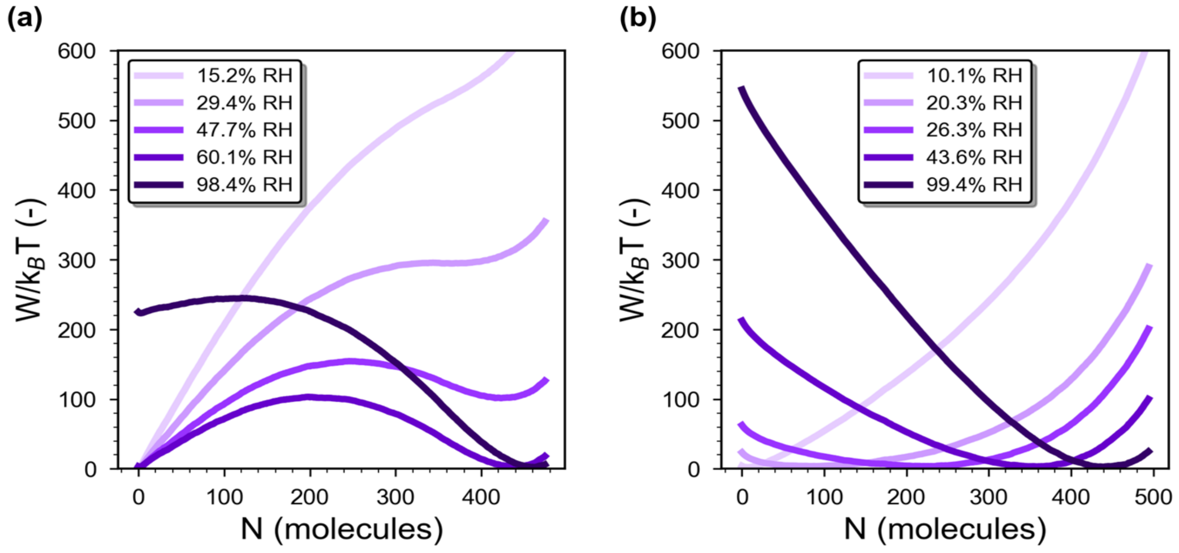

Figure S5. Grand potential free energy profiles of **(a)** MOF cg900153m_si_001 (bimodal) and **(b)** MOF YUBQET (unimodal).

3. Water Adsorption Mechanism

3.1. Adsorption Site Analysis

To understand the mechanism of water adsorption, it is essential to examine how the distribution of water molecules evolves with pressure. Since flat histogram Monte Carlo simulations are employed in this study, water molecule distributions in MOF should be computed through a weighted average of water configurations sampled across the canonical ensembles. We define this average configuration as "adsorption grid". This section begins by outlining the theoretical formulation for the adsorption grid, followed by its computational implementation. Under a fixed μ, V, T condition, the average loading is given by

$$< N >_{\mu VT} = \sum_{N=1}^{Nmax} \pi(N) \cdot N \tag{S20}$$

where $\pi(N)$ represents the normalized probability of the macrostate *N*. Since a macrostate encompasses multiple microstate configurations, $\pi(N)$ can be interpreted as the sum of the probabilities of all corresponding microstates. Therefore, Eq. (S20) can be reformulated as Eq. (S21):

$$\sum_{N=1}^{Nmax} \pi(N) \cdot N = \sum_{N=1}^{Nmax} \sum_{\forall i} \pi(C_i^N) \cdot N \tag{S21}$$

where C_i^N refers to the ith microstate configuration corresponding to the macrostate *N*. By applying the concept of conditional probability, the average loading can also be interpreted in terms of particle positions. Specifically, when the framework is divided into discrete grids, we can interpret *N* as the sum of the probabilities that each of the *N* particles occupies a specific grid position in the configuration C_i^N. When each grid can hold at most one molecule, this interpretation leads to Eq. (S22):

$$\sum_{N=1}^{Nmax} \sum_{\forall i} \pi(C_i^N) \cdot N = \sum_{N=1}^{Nmax} \sum_{\forall i} \pi(C_i^N) \cdot \sum_{\forall j} P(j|C_i^N) \tag{S22}$$

where *j* denotes the grid index within the framework. $P(j|C_i^N) = 1$ if a particle occupies grid *j* in configuration C_i^N; otherwise, $P(j|C_i^N) = 0$. Eq. (S22) can then be expressed in the form of Eqs. (S23), (S24), and (S25):

$$\sum_{N=1}^{Nmax} \sum_{\forall i} \pi(C_i^N) \cdot \sum_{\forall j} P(j|C_i^N)$$

$$= \sum_{N=1}^{Nmax} \sum_{\forall i} \sum_{\forall j} \pi(C_i^N) \cdot P(j|C_i^N) \tag{S23}$$

$$= \sum_{\forall j} \sum_{N=1}^{Nmax} \sum_{\forall i} P(C_i^N \cap j) \tag{S24}$$

$$= \sum_{\forall j} \sum_{\forall k} P(C_k \cap j) \tag{S25}$$

where k denotes the index of all possible microstate configurations under the given μ, V, T conditions, and the term $\sum_{\forall k} P(C_k \cap j)$ represents the expected value of the loading at grid j. Accordingly, Eq. (S25) precisely describes the spatial distribution of adsorbed water. This allows the distribution to be readily obtained by evaluating the joint probability of microstates and grid occupancy under the μVT ensemble using flat-histogram Monte Carlo simulations, where the sum over all grid points will equal the total average loading in the framework. It is emphasized that this method requires no additional computational cost. Once the MPD and the microstate configurations of each macrostate are available, the adsorption grid can be determined.

To ensure that each grid contains no more than one molecule, the framework is meshed into grids with dimensions smaller than 0.5 Å. 1,200 configurations are adopted for each macrostate when N≤20, and 500 configurations when N>20 to compute adsorption grids. These numbers are very small relative to the full configurational space of each macrostate, and the choice of the number of configurations may indeed possibly yield different absolute water adsorption distributions. However, the relative spatial preferences for water adsorption within the framework are expected to be qualitatively consistent.

3.2. Density Maps

Figure 6 of the main text clearly reveals that beyond the initial adsorption stage, additional water molecules in the bimodal MOF are adsorbed in a spatially dispersed manner throughout the pore network, while those in the unimodal MOF preferentially aggregate near pre-existing molecules, progressively expanding into denser, contiguous networks with increasing pressure. To assess the generality of this behavior, additional cross-sectional slices are also analyzed for both MOFs (RUGXOI01 and IWOKUC). Similar patterns, as shown in **Figure S6,** are observed. Furthermore, two structurally similar MOFs—ACAJIZ (bimodal; LCD = 6.19 Å; HoA = 39.03 kJ/mol) and QOVSOL (unimodal; LCD = 6.42 Å; HoA = 39.53 kJ/mol)—are compared. Their isotherms are presented in **Figure S7**. ACAJIZ again undergoes a dispersed-to-condensed transition, as shown in **Figure S8**, while QOVSOL exhibits that the clusters gradually expand locally with increasing pressures (**Figure S9**).

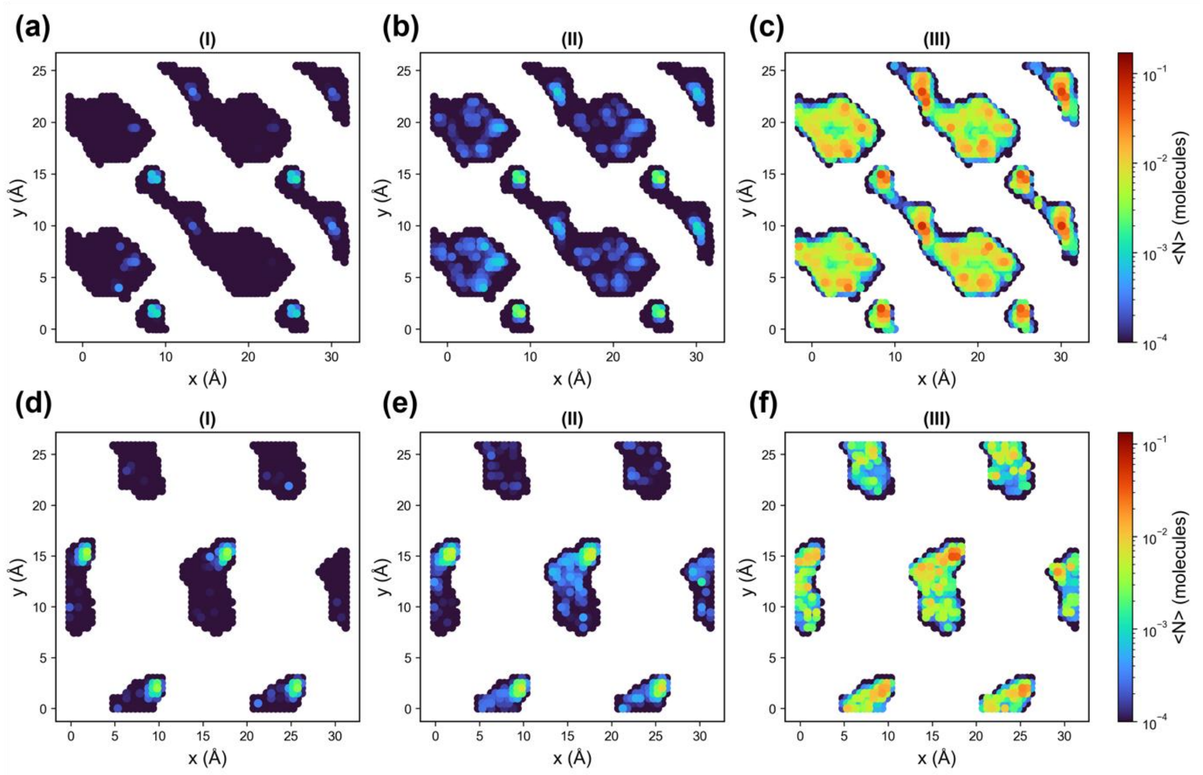

Figure S6. Cross-sectional water adsorption density maps of **(a-c)** RUGXOI01 at z-range of 4.9 to 5.4 Å and **(d-f)** IWOKUC at z-range of 7.45 to 7.95 Å. **(a-c)** and **(d-f)** show the water adsorption corresponding to the labeled pressures (I–III) of Figure 6(a,e) in the main text.

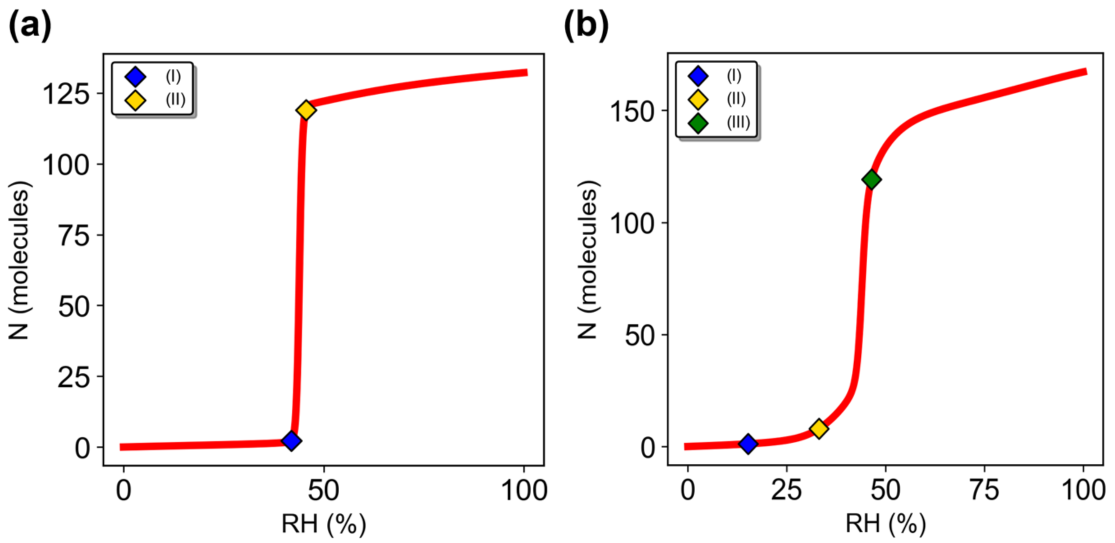

Figure S7. Water adsorption isotherms of **(a)** ACAJIZ (bimodal) and **(b)** QOVSOL (unimodal).

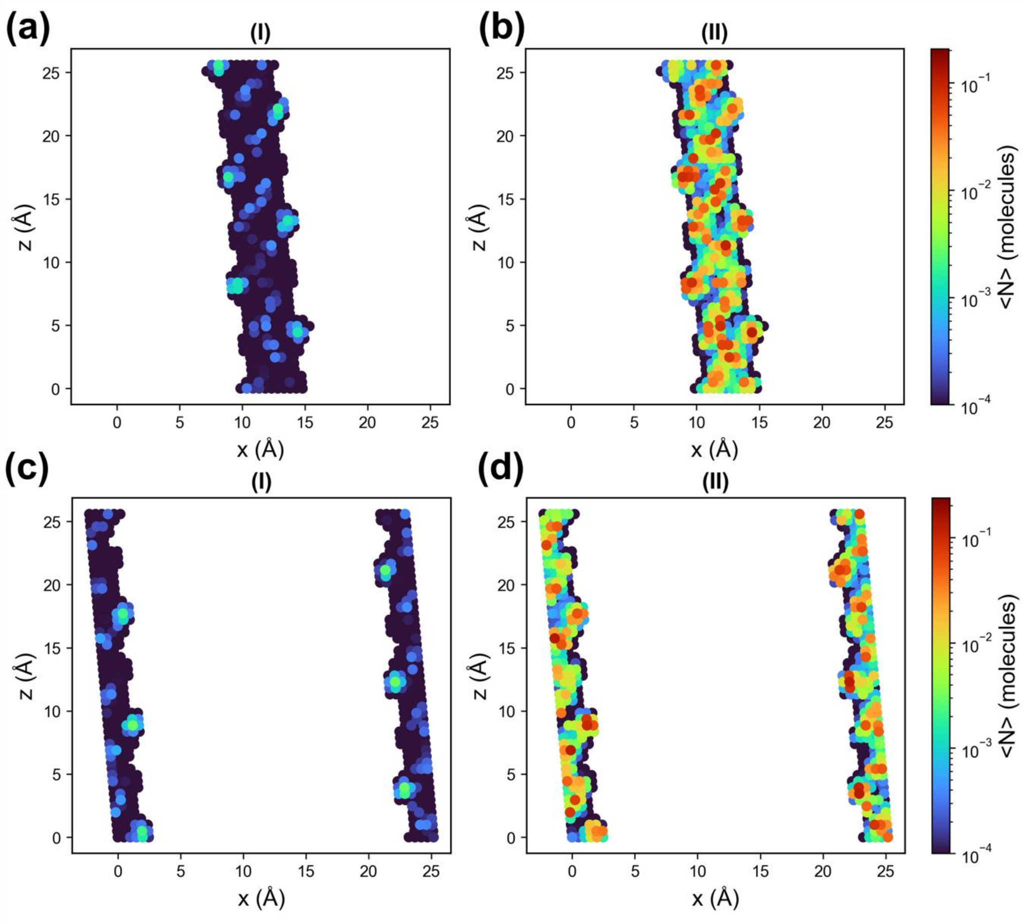

Figure S8. Cross-sectional water adsorption density maps of ACAJIZ at y-range of **(a-b)** 1.48 to 1.98 Å and **(c-d)** 4.93 to 5.43 Å. **(a-b)** and **(c-d)** show the water adsorption under labeled pressures (I–II) of **Figure S7(a)**.

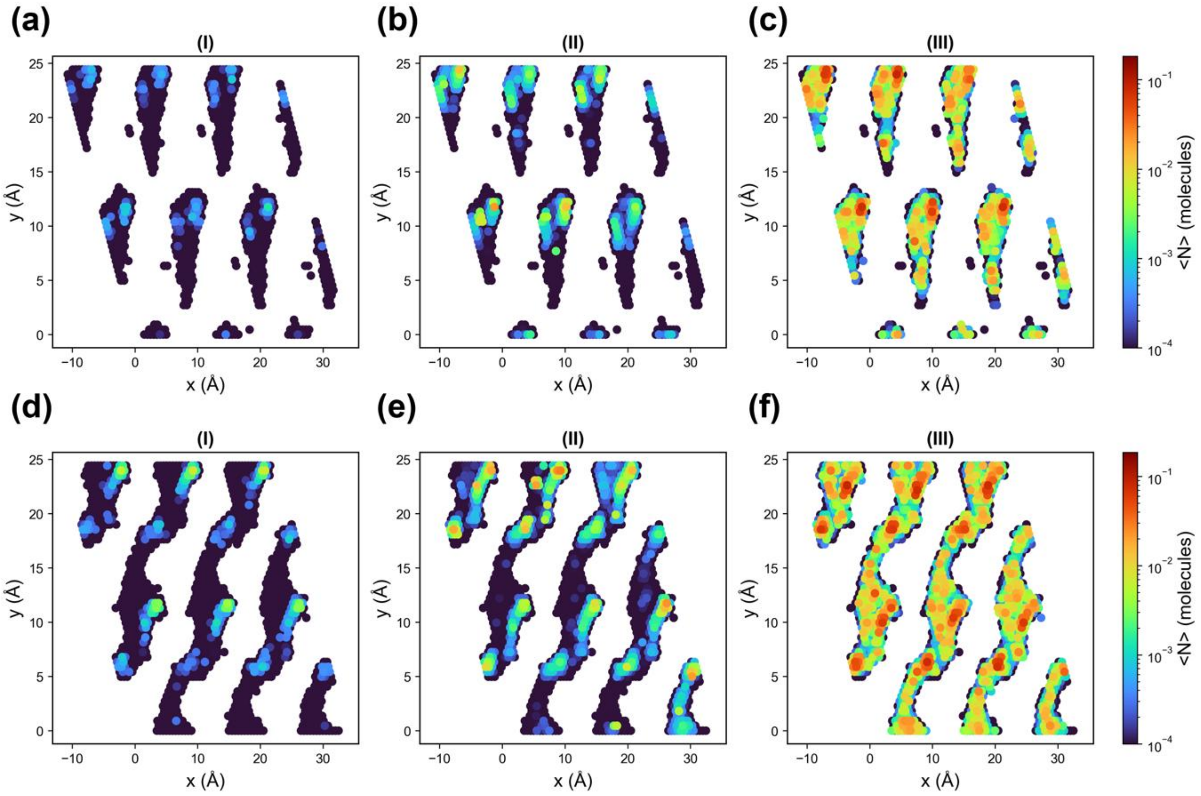

Figure S9. Cross-sectional water adsorption density maps of QOVSOL at z-range of **(a-c)** 2.49 to 3.49 Å and **(d-f)** 13.97 to 14.47 Å. **(a-c)** and **(d-f)** show the water adsorption under labeled pressures (I–III) of **Figure S7(b)**.

3.3. Radial Distribution Functions (RDFs) of Water Adsorbed in MOFs

This section demonstrates the O–O radial distribution functions for the bimodal MOF (i.e., RUGXOI01) and the unimodal MOF (i.e., IWOKUC) as discussed in the main text. For each relative humidity (RH), the RDF is calculated by averaging the RDFs at different water loadings and weighted by their macrostate probabilities. As discussed in the main text, these RDF trends are consistent with their corresponding density profiles and further support our proposed mechanistic distinction: in bimodal MOFs, water undergoes a phase transition, whereas in unimodal MOFs, adsorption proceeds via a gradual seeding and cluster growth mechanism.

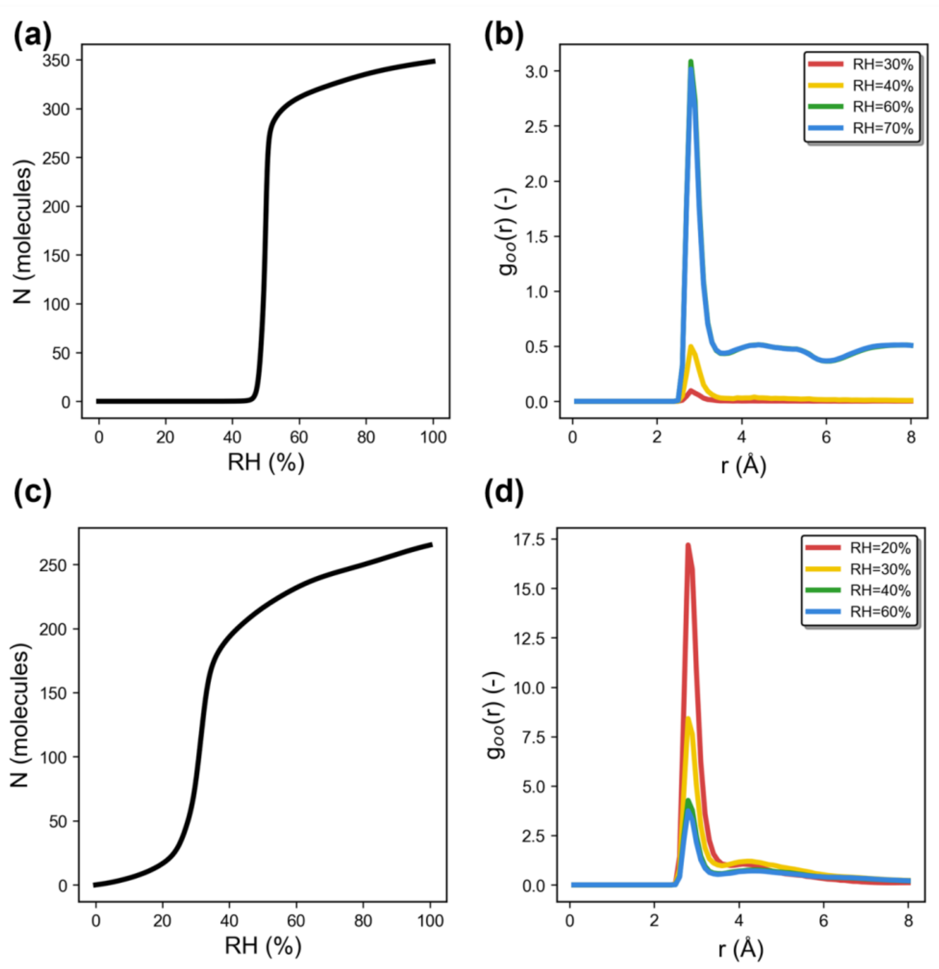

Figure S10. (a,c) Water adsorption isotherms and **(b,d)** the O–O radial distribution functions of adsorbed at varying RH values for the **(a,b)** bimodal MOF (i.e., RUGXOI01) and **(c,d)** the unimodal MOF (i.e., IWOKUC).

4. Hydrogen Bond Networks

This study also examines how different phase behaviors influence the hydrogen-bonding environment within MOFs. It should be noted that analyses of hydrogen bonds do not include those between MOF and water molecules. Adopting the criteria provided by Luzar et al.[11], a pair of water molecules is considered hydrogen bonded if the distance between their oxygen atoms is less than 3.5 Å and the angle O—H···O is smaller than 30°. Hydrogen bonding networks are typically analyzed under a fixed number of water molecules; however, the number of confined water molecules under saturation conditions varies. Therefore, we evaluate hydrogen bonding based on the most probable macrostate (i.e., the N with the highest probability) at saturation pressure.

The hydrogen bond number in the bulk phase is obtained via NPT molecular dynamics simulations at 298 K and 986 Pa. The simulation box contains 512 water molecules and has a length of 24.8 Å. The L-J interactions are truncated and shifted at a cutoff radius of 12 Å, while the tail correction is used. Our results are consistent with previous studies[12, 13] that used similar conditions and employed the similarly related TIP4P water model[14].

5. Vapor-Liquid Equilibrium (VLE) and Critical Properties

To determine the VLE of water in an adsorbent of interest, an elongated domain with multiple unit cells is simulated as depicted in **Figure S11**. Following the approach of Braun et al[15], the domain is initially configured with a partially filled liquid-like region and a vacant region to observe phase separation. As the simulation begins, water molecules will move from the liquid phase into the vapor region until equilibrium is established. The equilibrium properties are subsequently fitted to the scaling law[16] and the law of rectilinear diameters[17] to determine critical properties. All simulations are performed using the RASPA package[18]. Additional methodological details are described below.

5.1. Computational Details

The liquid-like configuration is first generated by performing an NVT simulation. The number of molecules, N, is estimated using the same method as N_{max} described in Section 2 of the main text. Following this, a GCMC desorption simulation is performed. The pressure for the GCMC simulation is determined by fitting the bulk VLE data to the Antoine equation (**Table S3**)[19]. The convergence criteria for both NVT equilibration and GCMC desorption steps require the standard deviation of energy and loading to be within 3% of their respective mean values. The simulation supercell is subsequently elongated along the direction that minimizes the interfacial area[15, 20]. Ten independent VLE simulations with different random seeds are conducted to produce relatively unbiased results. For each simulation, once both liquid and vapor densities exhibit clear plateaus upon equilibrium, the liquid and vapor phase densities are calculated by averaging the molecular distribution along the elongated axis over one thousand configurations (**Figure S12**). Specifically, the liquid density is obtained by averaging the N unit cells with the highest local densities over the cycles of the simulation, while the vapor density is derived from the M unit cells with the lowest densities. The integers N and M are structure-dependent and chosen to exclude interfacial regions while capturing representative bulk-like regions of each phase. These resulting cycle-averaged densities are then further averaged across the ten independent simulations to obtain the final equilibrium values. Simulation parameters for the selected MOFs are listed in **Table S4**.

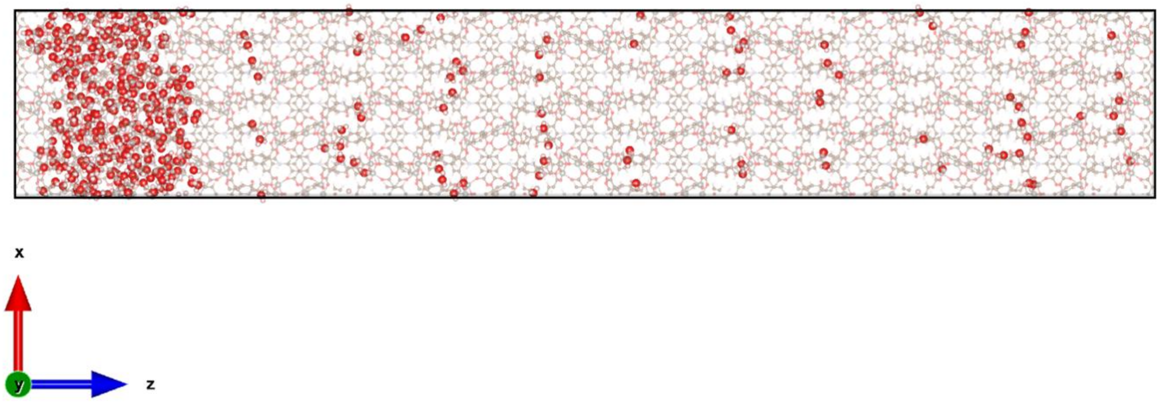

Figure S11. Elongated supercell composed of multiple unit cells used to model vapor–liquid equilibrium (VLE) of water in MOF (i.e., using ic502643m_si_008 as an example). Water molecules are shown as red and white spheres, and a distinct condensed phase is observed near the left end of the domain.

Table S3. Saturation water pressure under various temperatures determined by the Tip4p-Ew model.

T (K)	Saturation Pressure (Pa)
308	1866.71
318	3375.15
328	5855.10
338	9786.31
348	15817.26
358	24800.09
368	37826.72
378	56264.71
388	81792
398	116429.24

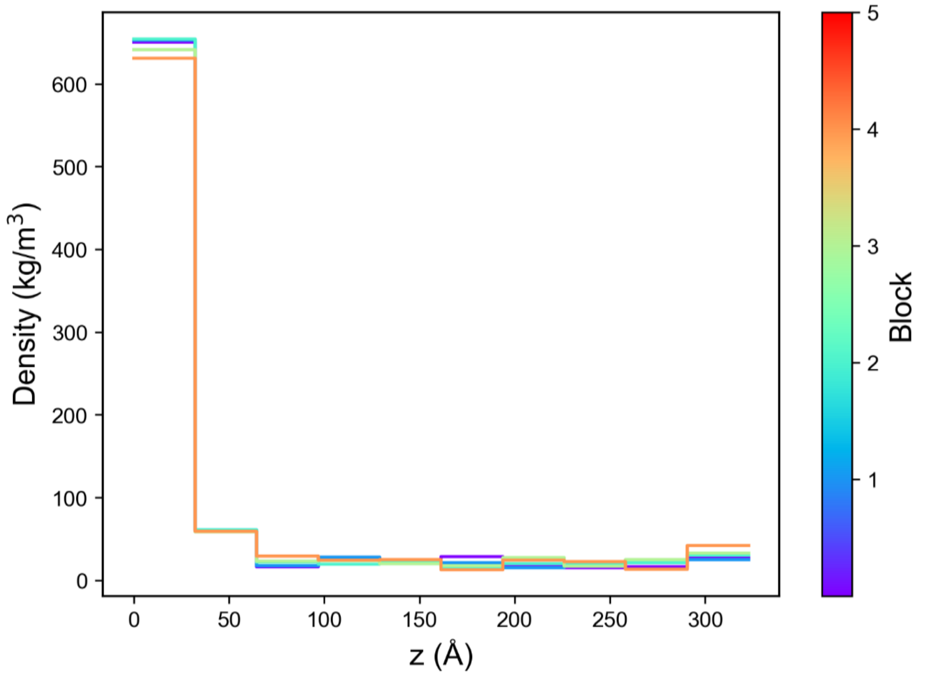

Figure S12. Corresponding density profile across the elongated direction in MOF ic502643m_si_008. Each segment represents the average density in a repeated unit along the *z*-axis. The colored lines represent independent block averages to assess convergence. A high-density liquid region is evident near z ~ 0–40 Å, followed by a low-density vapor region.

Table S4. Simulation parameters and equilibrium data. ρ_l and ρ_v represent liquid and vapor phase density, respectively.

Structure	Simulation box dimensions (unit cell #)	T (K)	Loading (molecules)	N (-)	M (-)	ρ_l (kg/m³)	ρ_v (kg/m³)
ACAJIZ	$1 \times 2 \times 50$	308	219	1	42	549.1	13.99
		318	211	1	42	377.38	20.77
		328	210	1	42	275.51	35.12
ic502643m_si_008	$3 \times 1 \times 10$	308	502	1	8	677.97	19.72
		318	488	1	8	608.58	26.58
		328	482	1	8	538.14	33.68
		338	478	1	8	450.17	39.33
		348	460	1	8	403.11	44.82
		358	460	1	8	370.08	47.19

5.2. Critical Properties

The critical parameters are determined by fitting the VLE data to the scaling law[16] and the law of rectilinear diameters[17], as shown in Eqs. (S26) and (S27), respectively.

$$\rho_l - \rho_g = B(T_c - T)^{\beta} \tag{S26}$$

$$\frac{\rho_l + \rho_g}{2} = \rho_c + A(T - T_c) \tag{S27}$$

In these two equations, T_c and ρ_c represent the critical temperature and density, A and B are the fitting parameters, and β is the universal critical exponent. Since the confined fluid does not behave as a fully three-dimensional bulk fluid, β is treated as an adjustable fitting parameter rather than being fixed at 0.325, which corresponds to three-dimensional Ising universality[21]. The calculated critical parameters are shown in **Table S5**.

Table S5. Critical properties of water adsorbed in ACAJIZ and ic502643m_si_008.

Structure	T_c (K)	ρ_c (kg/m^3)
ACAJIZ	333.06	116.95
ic502643m_si_008	391.97	137.12

6. Step Pressure

This study evaluates the relationship between step pressure and two novel descriptors (i.e., hydrophilicity index (i_H)[22] and the newly developed connectivity index (i_c)). Both descriptors rely on the construction of the energy grid, which is generated following the methodology described by Xu et al[23]. It is emphasized that periodic boundary conditions must be applied when evaluating 3 Å neighborhoods for the calculation of i_c.

This section provides the supporting results. **Figure S13** shows the correlation between step pressure and i_H, adapted from the study of Nguyen et al.[22] **Figure S14** compares the correlation between step pressure and i_c of bimodal and unimodal MOFs. **Figure S15** illustrates the sensitivity of i_c to different energy thresholds.

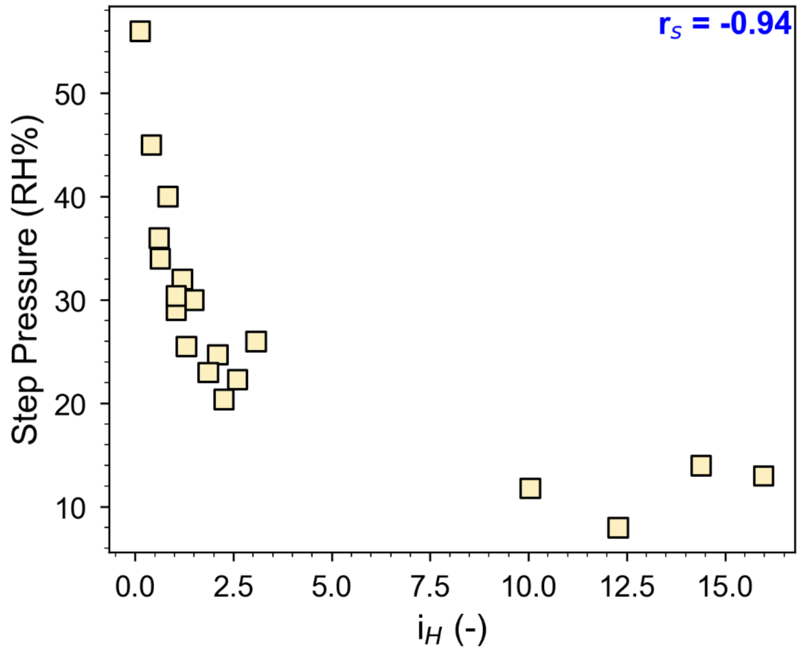

Figure S13. Correlation between step pressure and hydrophilicity index (i_H). The data shown in this figure is adapted from the study of Nguyen et al.[22].

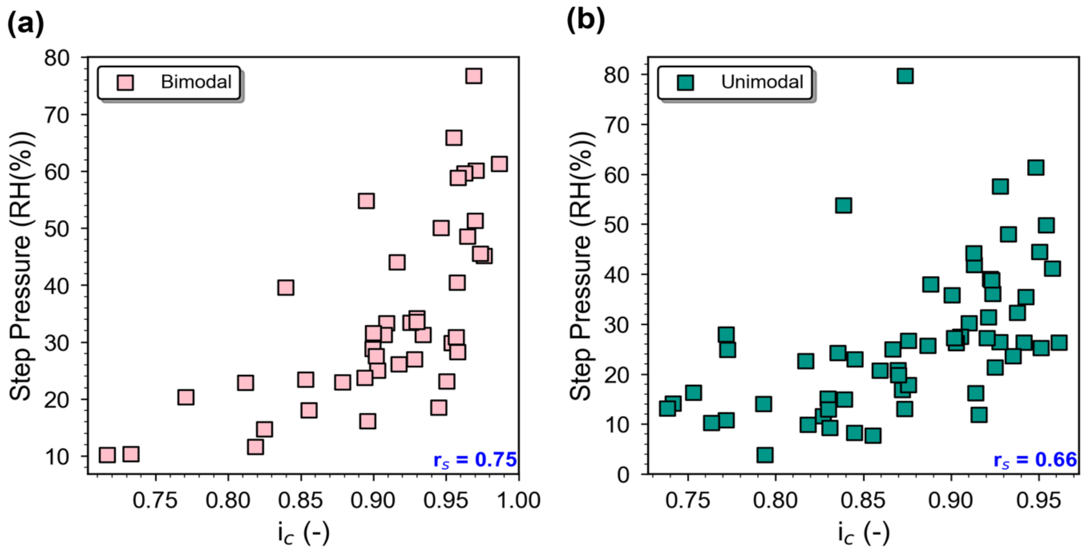

Figure S14. Correlation between step pressure and connectivity index (i_c) of **(a)** bimodal and **(b)** unimodal MOFs.

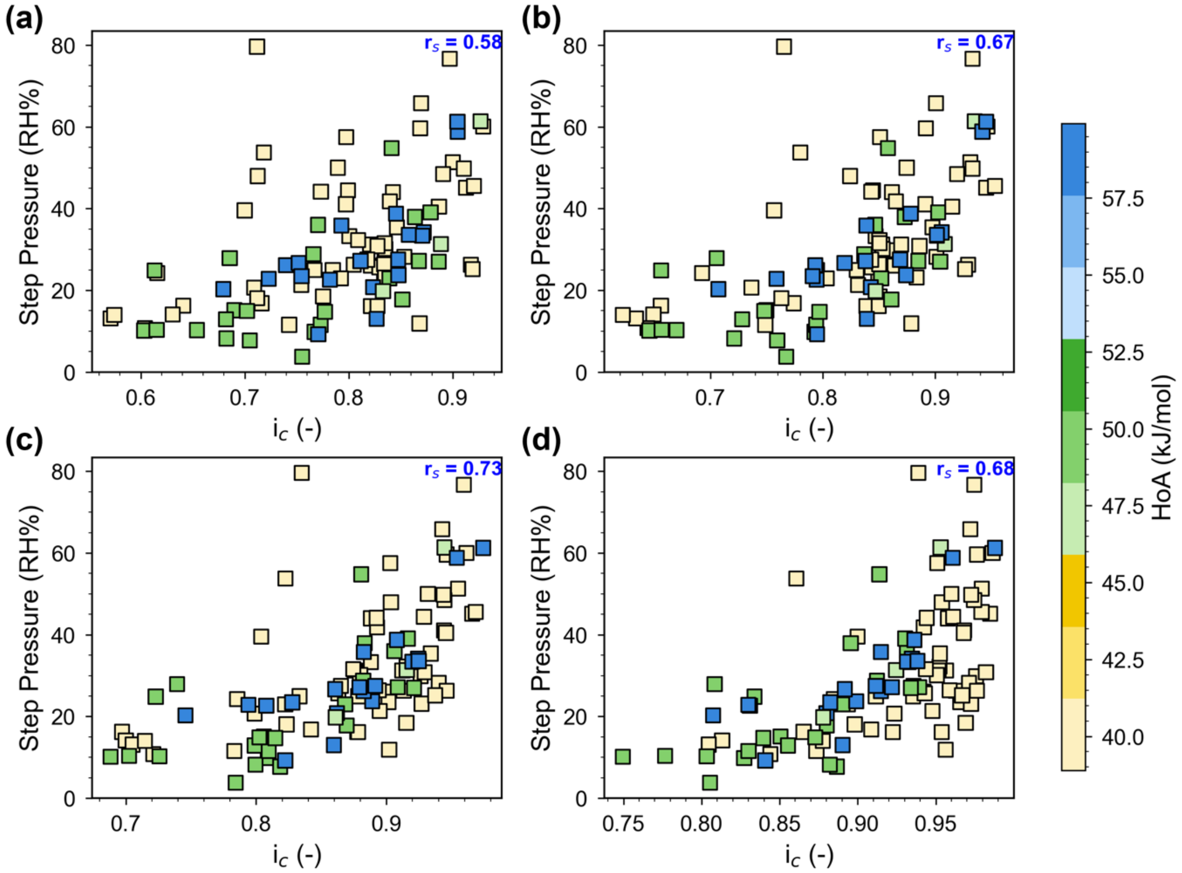

Figure S15. The correlation between i_c and step pressure with the selected energy threshold of adsorption sites of **(a)** –3600K, **(b)** –4050K, **(c)** –4500K, and **(d)** –4950K. Spearman correlation coefficient r_s increases from 0.58 at –3600 K to 0.67 at –4050 K, peaks at 0.73 with –4500 K, and declines to 0.68 at –4950 K. It indicates that i_c remains a robust predictor to inform step pressure, despite its sensitivity to the choice of energy threshold.

7. References

(1) Witman, M. Flat-Histogram Monte Carlo as an Efficient Tool To Evaluate Adsorption Processes Involving Rigid and Deformable Molecules. *J. Chem. Theory Comput.* **2018**, *14* (12), 9. DOI: 10.1021/acs.jctc.8b00534.

(2) Witman, M.; Wright, B.; Smit, B. Simulating Enhanced Methane Deliverable Capacity of Guest Responsive Pores in Intrinsically Flexible MOFs. *J. Phys. Chem. Lett.* **2019**, *10* (19), 5929–5934. DOI: 10.1021/acs.jpclett.9b02449.

(3) Errington, J. R. Direct calculation of liquid–vapor phase equilibria from transition matrix Monte Carlo simulation. *J. Chem. Phys.* **2003**, *118* (22), 9915–9925. DOI: 10.1063/1.1572463.

(4) Shen, V. K.; Errington, J. R. Metastability and Instability in the Lennard-Jones Fluid Investigated by Transition-Matrix Monte Carlo. *J. Phys. Chem. B* **2004**, *108* (51), 19595–19606. DOI: 10.1021/jp040218y.

(5) Datar, A.; Witman, M.; Lin, L. C. Monte Carlo simulations for water adsorption in porous materials: Best practices and new insights. *AlChE J.* **2021**, *67* (12), e17447. DOI: 10.1002/aic.17447.

(6) Mayo, S. L.; Olafson, B. D.; Goddard, W. A. DREIDING: a generic force field for molecular simulations. *J. Phys. Chem.* **1990**, *94* (26), 8897–8909. DOI: 10.1021/j100389a010.

(7) Rappé, A. K.; Casewit, C. J.; Colwell, K.; Goddard III, W. A.; Skiff, W. M. UFF, a full periodic table force field for molecular mechanics and molecular dynamics simulations. *J. Am. Chem. Soc.* **1992**, *114* (25), 10024–10035. DOI: 10.1021/ja00051a040.

(8) Horn, H. W.; Swope, W. C.; Pitera, J. W.; Madura, J. D.; Dick, T. J.; Hura, G. L.; Head-Gordon, T. Development of an improved four-site water model for biomolecular simulations: TIP4P-Ew. *J. Chem. Phys.* **2004**, *120* (20), 9665–9678. DOI: 10.1063/1.1683075.

(9) Tester, J. W.; Modell, M. *Thermodynamics and its Applications*; Prentice Hall, 1997.

(10) Ott, J. B.; Boerio-Goates, J. The Equilibrium Condition Applied to Phase Equilibria. In *Chemical Thermodynamics: Principles and Applications*, Ott, J. B., Boerio-Goates, J. Eds.; Academic Press, 2000; pp 383–433.

(11) Luzar, A.; Chandler, D. Effect of Environment on Hydrogen Bond Dynamics in Liquid Water. *Phys. Rev. Lett.* **1996**, *76* (6), 928–931. DOI: 10.1103/PhysRevLett.76.928.

(12) Xu, H.; Stern, H. A.; Berne, B. J. Can Water Polarizability Be Ignored in Hydrogen Bond Kinetics? *J. Phys. Chem. B* **2002**, *106* (8), 2054–2060. DOI: 10.1021/jp013426o.

(13) Oktavian, R.; Goeminne, R.; Glasby, L. T.; Song, P.; Huynh, R.; Qazvini, O. T.; Ghaffari-Nik, O.; Masoumifard, N.; Cordiner, J. L.; Hovington, P.; et al. Gas adsorption and framework flexibility of CALF-20 explored via experiments and simulations. *Nat. Commun.* **2024**, *15* (1), 3898. DOI: 10.1038/s41467-024-48136-0.

(14) Jorgensen, W. L.; Chandrasekhar, J.; Madura, J. D.; Impey, R. W.; Klein, M. L. Comparison of simple potential functions for simulating liquid water. *J. Chem. Phys.* **1983**, *79* (2), 926–935. DOI: 10.1063/1.445869.

(15) Braun, E.; Chen, J. J.; Schnell, S. K.; Lin, L.-C.; Reimer, J. A.; Smit, B. Nanoporous Materials Can Tune the Critical Point of a Pure Substance. *Angew. Chem. Int. Ed.* **2015**, *54* (48), 14349–14352. DOI: 10.1002/anie.201506865.

(16) Rowlinson, J. S.; Widom, B. *Molecular theory of capillarity*; Courier Corporation, 2013.

(17) Rowlinson, J.; Swinton, F. Liquids and Liquid Mixtures, Butter-worths. London: 1982.

(18) Dubbeldam, D.; Sofía, C.; E., E. D.; and Snurr, R. Q. RASPA: molecular simulation software for adsorption and diffusion in flexible nanoporous materials. *Mol. Simul.* **2016**, *42* (2), 81–101. DOI: 10.1080/08927022.2015.1010082.

(19) Vega, C.; Abascal, J. L. F.; Nezbeda, I. Vapor-liquid equilibria from the triple point up to the critical point for the new generation of TIP4P-like models: TIP4P/Ew, TIP4P/2005, and TIP4P/ice. *J. Chem. Phys.* **2006**, *125* (3), 034503. DOI: 10.1063/1.2215612.

(20) Janeček, J.; Krienke, H.; Schmeer, G. Inhomogeneous Monte Carlo simulation of the vapor-liquid equilibrium of benzene between 300 K and 530 K. *Condens. Matter Phys.* **2007**.

(21) Emelianova, A.; Basharova, E. A.; Kolesnikov, A. L.; Arribas, E. V.; Ivanova, E. V.; Gor, G. Y. Force Fields for Molecular Modeling of Sarin and its Simulants: DMMP and DIMP. *J. Phys. Chem. B* **2021**, *125* (16), 4086–4098. DOI: 10.1021/acs.jpcb.0c10505.

(22) Nguyen, H. L.; Darù, A.; Chheda, S.; Alawadhi, A. H.; Neumann, S. E.; Wang, L.; Bai, X.; Alawad, M. O.; Borgs, C.; Chayes, J. T.; et al. Pinpointing the Onset of Water Harvesting in Reticular Frameworks from Structure. *ACS Cent. Sci.* **2025**, *11* (5), 665–671. DOI: 10.1021/acscentsci.4c01878.

(23) Xu, Z.-X.; Wang, Y.-M.; Lin, L.-C. Connectivity Analysis of Adsorption Sites in Metal–Organic Frameworks for Facilitated Water Adsorption. *ACS Appl. Mater. Interfaces* **2023**, *15* (40), 47081–47093. DOI: 10.1021/acsami.3c10710.
